# Deciphering microbial interactions using a label-free microbead sorting approach

**DOI:** 10.1093/ismeco/ycag058

**Published:** 2026-03-13

**Authors:** Sagarika B Govindaraju, Daan H de Groot, Rinke J van Tatenhove-Pel

**Affiliations:** Department of Biotechnology, Faculty of Applied Sciences, TU Delft, Delft 2629 HZ, The Netherlands; Department of Computational Systems Biology, Biozentrum, University of Basel, Basel 4056, Switzerland; Department of Biotechnology, Faculty of Applied Sciences, TU Delft, Delft 2629 HZ, The Netherlands

**Keywords:** microbial interactions, microbial consortia, microdroplet cultivation, label-free detection

## Abstract

Microorganisms form communities, and their interactions shape the function and stability of these communities. Understanding these interactions can aid in revealing ecosystem dynamics, enhancing community function, and informing the design of synthetic consortia for industrial applications. Deciphering microbial interactions is challenging due to the difficulty of culturing natural microorganisms and the exponential increase in experiments with expanding consortium size. One approach to improving culturing throughput is the use of microcompartments such as agarose microbeads. Microbead-based techniques enable the generation of large numbers of picolitre-sized compartments, facilitating high-throughput, parallel studies of microbial sub-communities. However, the existing microbead-based techniques for deciphering microbial interactions are dependent on single-culture isolates of consortium members and/or labelling of consortium members with fluorescent markers via genetic engineering. We developed a microbead-based, label-free method that eliminates the requirement of single-cell isolates to predict microbial interactions. Our method involves an isolation-independent manner of microbead inoculation with different sub-communities and microbead sorting to separate sub-communities based on growth. Using a probabilistic model, we predict interactions based on cell concentrations and relative abundances in the inoculum and after microbead sorting. We successfully predicted pairwise interactions in two three-member consortia. Additionally, we computationally showcased the validity of our approach for predicting pairwise interactions in larger consortia.

## Graphical Abstract

**Graphical Abstract:**
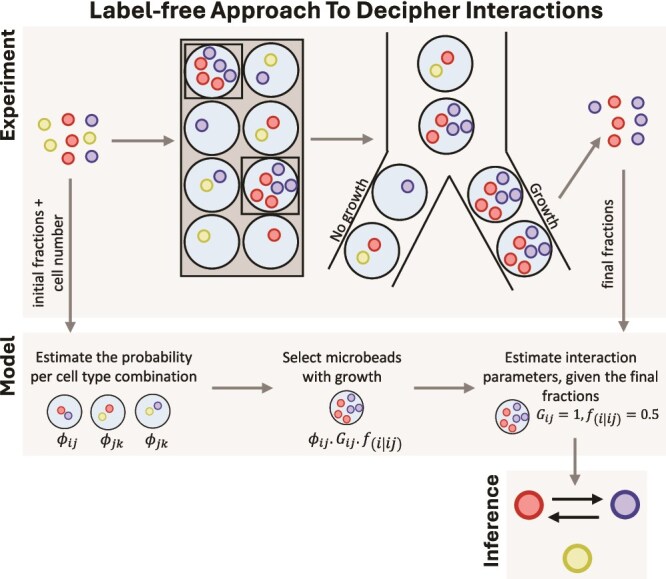

## Introduction

Microorganisms exist in communities in diverse environments such as soil, oceans, and human bodies. Interactions between the microorganisms shape the function and stability of these communities and thereby also the ecosystems [1–3]. By deciphering microbial interactions, we can gain deeper insight into ecosystem dynamics, identify ways to improve community function, and even derive design principles to develop synthetic consortia for industrial applications.

To decipher microbial interactions, one can use isolation-independent and isolation-dependent approaches. Isolation-independent approaches determine interactions without needing single-cell isolates of community members. They predict interactions, to the level of metabolites exchanged, by using inter- and intra-species metabolic fluxes. They leverage advanced imaging and/or omics technologies to analyse community composition [1, 4, 5]. However, these approaches postulate possible interactions, and it is challenging to experimentally verify the deduced interactions. Alternatively, isolation-dependent approaches predict types of interactions [1, 4, 5] (e.g. cooperation, inhibition, competition) in natural microbial communities by constructing and studying synthetic mimic communities from culturable micro-organisms [1, 4, 5]. Isolation-dependent approaches directly use growth and species abundance-based data to decipher interactions. However, given the challenge of obtaining single culture isolates of microorganisms from environmental samples, typically, synthetic communities that mimic the natural communities are constructed, but these synthetic communities often fail to fully capture the complex interactions in natural communities [1, 4–7]. In addition, as the size of a microbial consortium increases, the scale of experiments grows exponentially, as these approaches necessitate culturing of all possible combinations of strains and species.

One way to enhance culturing throughput is to utilise microcompartments. Microdroplet-based techniques enable the generation of large numbers of water-in-oil emulsions in a small volume (~10^7^ microbeads with a diameter of 40 μm in 1 ml), allowing high-throughput parallel studies of sub-communities. The applicability of microcompartments to predict microbial interactions has been previously demonstrated [7–9]. Growth and relative abundances in microdroplets were determined by labelling the different cell types with fluorescent markers and monitoring the growth of each sub-community. Therefore, these methods still require single culture isolates of the community members and genetic accessibility of the isolates to label them with fluorescent markers [7, 9].

In this study, we developed an agarose microbead-based, isolation-independent, and label-free method to predict microbial interactions. We inoculate microbeads with different sub-communities and sort microbeads with sub-communities based on growth. We predict interactions based only on the cell concentration and relative abundances in the inoculum and after microbead sorting, using a Bayesian inference model. Measurement errors in the abundance data were explicitly modelled to achieve more accurate estimations of interaction parameters. Our results show that we can predict pairwise interactions in two different three-member consortia. Furthermore, we computationally validated our method to predict interactions in larger consortia with diverse interactions.

## Materials and methods

### Strains, media, and culture conditions

Table 1 describes the strains that were used in this study. *Lactococcus cremoris* strains were grown in chemically defined medium for prolonged cultivations (CDMpc) [15]*.* For growth on casein as a nitrogen source, 2 g/L of casein sodium salt (from bovine milk, C8654, Sigma-Aldrich, Saint Louis, MO, USA) was added to the medium instead of the amino acids (CDMpc_cas). For growth in CDMpc with limiting amounts of amino acids and glucose, 1% of the amino acid concentration in CDMpc and 0.02% (wt/v) glucose were added to CDMpc_cas (CDMpc_cas,aa,glu). For preparation of agar plates, 2% (wt/v) sterilised agar was added to CDMpc +0.5% (wt/v) trehalose or M17 (CM0817, Oxoid, Ireland) + 0.5% (wt/v) glucose. For long-term storage at −80°C, glycerol was added to overnight cultures of *L. cremoris* to obtain a final concentration of 25% (v/v).

**Table 1:** Strains used in this study.

**Strain**	**Genotype**	**Description**	**Reference**
**NZ9000 Glu** ^**−**^ **Lac**^**+**^	*L. cremoris* NZ9000*ΔglkΔptnABCD* carrying pMG8020 (derivative of pLP712, carrying *lacFEGABCD*) and contains a 657-bp deletion in *ptcBA*.	Uses the galactose moiety of lactose as a carbon source and secretes the glucose moiety. Cannot use casein as a nitrogen source.	[10]
**MG610**	*L. cremoris* MG1363 with two copies of *prtMP* integrated into the genome, erythromycin resistant.	Uses glucose as a carbon source and casein or amino acids as a nitrogen source.	[11]
**NZ5500**	*L. cremoris* MG5267 with a loxP-ery-usp45p-melA-loxP fragment integrated into the genome, erythromycin resistant.	Uses lactose and glucose as carbon sources. Cannot use casein as a nitrogen source.	[12]
**MG1363**	Wildtype.	Uses glucose as a carbon source. Cannot use casein as a nitrogen source.	[13]
**MG1363-GFP**	*L. cremoris* MG1363 carrying pSEUDO::*P_usp45_-gfp.*	Expresses GFP. Uses glucose as a carbon source. Cannot use casein as a nitrogen source.	[14]

We grew the pre-cultures of *L. cremoris* NZ9000 Glu^−^ Lac^+^ and *L. cremoris* NZ5500 in CDMpc +0.09% (wt/v) lactose, and the pre-cultures of *L. cremoris* MG610, *L. cremoris* MG1363-GFP, and *L. cremoris* MG1363 in CDMpc +0.09% (wt/v) glucose. Pre-cultures were incubated at 30°C statically for at least 16 h. Co-cultures or monocultures of the strains when grown in CDMpc_cas,aa,glu + 0.5% (wt/v) lactose, were incubated for at least 40 h at 30°C statically. The cultures were de-coagulated after growth by adding sodium hydroxide to increase the pH to no more than 7.

We determined the relative proportions of *L. cremoris* MG1363 and *L. cremoris* MG1363-GFP in the inoculum for emulsions using flow cytometry. We determined the proportions of these two strains after microbead sorting in phosphate-buffered saline (PBS) by vortexing the solution with microbeads and plating on M17 + 0.5% (wt/v) glucose agar plates. After incubation at 30°C for at least 40 h, we counted the resulting fluorescent and non-fluorescent colonies by illuminating the plate with Safe Imager 2.0 Blue-light Transilluminator (Invitrogen, MA, USA).

To determine the fractions of different cell types in suspension cultures, before and after growth, we plated 100–200 cells (based on measured cell concentrations) on agar plates containing CDMpc +0.5% (wt/v) trehalose.

To determine the fraction of different cell types after microbead sorting, we vortexed the sort-tubes containing microbeads and phosphate buffer saline (PBS) and plated the suspension on agar plates with CDMpc +0.5% (wt/v) trehalose, which supports the growth of all strains. The plates were incubated at 30°C for at least 40 h. From each trehalose plate, 48–95 randomly picked colonies were individually inoculated in four different media: (i) CDMpc +0.5% (wt/v) lactose, (ii) CDMpc +0.5% (wt/v) lactose +10 μg/ ml erythromycin, (iii) CDMpc_cas + 0.5% (wt/v) glucose and, (iv) CDMpc +0.5% (wt/v) glucose. Each colony was assigned a number, and the same numbered colony was inoculated in all four media to allow direct comparison. The ability of each colony to grow on these media was used to distinguish the strains as follows:

1. Colonies that grow on media 1, 2 and 4 are *L. cremoris* NZ5500.
2. Colonies that grow only on medium 1 are *L. cremoris* NZ9000 Glu^−^ Lac^+^.
3. Colonies that grow on media 3 and 4 are *L. cremoris* MG610.
4. Colonies that grow only on medium 4 are *L. cremoris* MG1363.

Colonies that deviated from these described growth profiles were excluded from the analysis, which were no more than 4% of the screened colonies.

### Analytical methods

Optical density (OD) was measured at 660 nm using a Libra S11 spectrophotometer (Biochrom, Cambridge, United Kingdom). Cell concentration and microbeads were measured using a BD Accuri™ C6 Plus flow cytometry (BD Biosciences, San Jose, CA, USA). Concentrations of sugars, organic acids, and ethanol were measured via HPLC analysis on an Agilent 1260 HPLC (Agilent Technologies, CA, USA), equipped with a Bio-Rad HPX 87H column (Bio-Rad, CA, USA). Detection was performed by an Agilent refractive index detector and an Agilent 1260 VWD detector. The size and volume distributions of the microbeads in oil were determined by imaging the emulsions using a Zeiss Axio Imager Z1 (Carl Zeiss AG, Jena, Germany) and an AxioCam HRm Rev3 detector (60 N-C 1″ × 1.0; Carl Zeiss AG) using the lateral magnification objective ×20. Details on the estimation of the average microbead size are described in SI: 1.

### Preparation of microbeads

Agarose microbeads in oil were prepared with a water phase and an oil phase (Novec HFE 7500 fluorinated oil, 3 M, Maplewood, MN, USA) containing 0.2% PicoSurf 1 surfactant (Sphere Fluidics, Cambridge, UK). The water phase contained CDMpc_cas or CDMpc_cas,aa,glu with 1% (wt/v) agarose with ultra-low gelling temperature (Type IX-A, A2576, Sigma-Aldrich, Saint Louis, MO, USA) and, when required, cells. At this low agarose concentration, we do not expect significant limitations in diffusion of nutrients.

Cells from pre-cultures were harvested when the OD_660_ was between 0.15–0.40. The cells were centrifuged (5 min 3000 × *g*), washed with PBS, and the cell concentration was measured. The cells were diluted based on inoculation Poisson rate $\lambda$ (0.02 or 0.3) in CDMpc_cas or CDMpc_cas,aa,glu + 0.5% (wt/v) lactose. To prepare the emulsions, 300 μL of the water phase was mixed with 700 μL of the oil phase with the surfactant using a T10 basic ULTRA TURRAX homogeniser with an S10N-5G dispersing element (IKA, Staufen, Germany) at speed 2.5 for 5 min. The microbeads had an average volume of 49.65 pL ± 8.02 pL (diameter of 44.15 μm ± 2.37 μm, n = 8, Fig. S1). The agarose was solidified by placing the emulsions on ice for at least 20 min. The emulsions were incubated statically at 30°C for at least 40 h. After incubation, the microbeads were separated from the oil phase by adding 1.2 ml PBS and 1.5 ml perfluorooctanol (PFO, Alfa Aesar, Ward Hill, MA, USA) to 1 ml of the emulsion and gently mixing. The microbeads partitioned to the water phase, i.e. PBS, which was gently pipetted out.

### Sorting of microbeads

Before measuring on the flow cytometer or the FACS, the microbeads were filtered with a 40 μm mesh (pluriStrainer, PET-mesh). Microbeads smaller than 40 μm were further analysed. The forward scatter, side scatter, and the green fluorescence signal were measured using the 488 nm laser. We analysed the microbeads with a flow cytometer and sorted them with the BD FACSAria™ II SORP Cell Sorter (BD Biosciences, San Jose, CA, USA) operated with the FACSFlow™ software (BD Biosciences) using a 130 μm nozzle. The green fluorescence signal was detected through a 545/28 nm or a 530/30 nm bandpass filter. Microbeads were sorted based on the sorting thresholds depicted in Fig. S5 (step 4) into tubes with 800 μL PBS or on agar plates with CDMpc +0.5% (wt/v) trehalose. FlowJo™ v10.10 software (BD Life Sciences) was used for data analysis. Details about the gating strategy are shown in Fig. S5.

### Probabilistic inference model

We used the data with a Bayesian inference scheme to determine the interaction parameters in a simple model of microbial interaction.

### Available data

For each experiment, the available data are measurements of the cell-type frequencies before and after the growth phase. These frequencies are measured by taking a random sample of ~96 cells and determining the cell-type. We collect these data in a count vector $\overrightarrow{d}(0)$ for the initial counts, and $\overrightarrow{d}(T)$ for the final counts. These counts do not exactly reflect the cell-type proportions, which is why we explicitly model the counts $\overrightarrow{d}(0)$ as a multinomial sample of the true cell-type proportions using an experimental preparation precision value (Fig. S7) $\overrightarrow{p}(0)$. See SI: 6 and Fig. S8 for the mathematical details.

### Modelling the distribution of cells over the microbeads

Given the initial cell-type proportions $\overrightarrow{p}(0)$, we now model the fractions of microbeads with different cell-type compositions. This consists of two steps. We first model the fraction of microbeads with a certain number of cells. The cell-number in the microbeads is assumed to be Poisson-distributed with a rate $\lambda$ that can be set experimentally. Then, for each microbead with a certain number of cells, we model the cell-type composition as a multinomial distribution where the probability of a cell being of a certain strain is given by the proportions $\overrightarrow{p}(0)$. Together, this gives us the expected fraction of microbeads that have a certain cell-type composition. For example, we denote by ${\phi}_{ij}$ the fraction of microbeads that have one cell of cell-type $i$ and one cell of cell-type $j$. Importantly, in the experiments, we always choose Poisson-rates $\lambda$ that are small enough such that the number of microbeads with more than 2 cells is low. For that reason, we will ignore contributions of microbeads with more than 2 cells. See SI: 6 and Fig. S8 for the mathematical details.

### Proposed model of cell-type interactions in microbeads

Now that we have the fraction of microbeads with a certain cell-type composition (${\phi}_{ij}$), we need to model the number of offspring cells that a cell combination in such microbeads will give rise. We do this using two parameters per cell-type combination: ${G}_{ij}$ and ${f}_{i\mid ij}$. Here, ${G}_{ij}$ gives the average final number of cells in a microbead that was inoculated with an $i,j$-pair, and ${f}_{i\mid ij}$ denotes the fraction of these cells i.e. of type $i$. The eventual number of cells of type $i$ in this microbead is thus given by ${G}_{ij}{f}_{i\mid ij}$. Note here that, since our measurements only reveal relative cell-type abundances, we cannot determine absolute values for ${G}_{ij}$ from our experiments. Therefore, we will always report relative growth factors which are normalized with respect to a reference cell-type combination. Using this model and the expected fraction of different microbeads, we can now predict the eventual number of cells for each cell-type, and thus the final cell-type proportions. These cell-type proportions are again measured by taking a random sample giving rise to the count vectors $\overrightarrow{d}(T)$. See SI: 6 and Fig. S8 for the mathematical details.

### Incorporation of prior information

We are ultimately interested in inferring these interaction parameters ${G}_{ij}$ and ${f}_{i\mid ij}$, and in SI: 6, we explain how we approximate the posterior probability distribution for these parameters, so that we can report the maximum posterior probability intervals and a 68% posterior probability interval (ppi). To get these posteriors, we need to pick prior probability distributions for these parameters (see SI: 6 for more details). In short, when we do not have prior information on the values of these parameters (e.g. in the experiment with the first consortium with strains A, B and C), we take the fraction ${f}_{i\mid ij}$ to be uniform on the unit interval, and ${G}_{ij}$ to be uniform in log-scale: $P\left({G}_{ij}\right)\propto 1/{G}_{ij}$ (Tables S1 and S2). The latter can be intuitively understood by realizing that it gives equal weight to this relative growth factor being near 0.5 as it being near 2. If we have more information on these parameters (e.g. in the experiment with the second consortium with strains A, B and C^*^), we capture this by specifying the mean and variance of a Dirichlet prior for ${f}_{i\mid ij}$ and a log-normal prior for ${G}_{ij}$ (Tables S1 and S2).

### Computational validation using simulated data

To validate the inference model for larger consortia, we created simulated datasets for consortia with up to six members. Detailed implementation of the computational validation is described in SI:7. Two types of consortia were designed: (i) consortia where none of the cell types could grow independently, i.e. no isolated growth, and (ii) consortia where one or two cell types were capable of isolated growth. In scenarios where there is a negative interaction, we assume that both the cell types exhibit isolated growth, and depending on the direction of the interaction, we expect at least one of the cell types to grow. We defined growth until medium depletion relative to the reference cell type pair, which should always grow until medium depletion.

One-sided t-tests were performed to test for significant increases in one condition compared to another, whereas two-sided t-tests were used to test for significant differences in either direction.

All computational results were obtained using Python code, which is freely available on GitHub at https://github.com/Sagarikabg/Deciphering_Microbial_Interactions.git.

## Results

### Method to decipher microbial interactions

**Figure 1:**
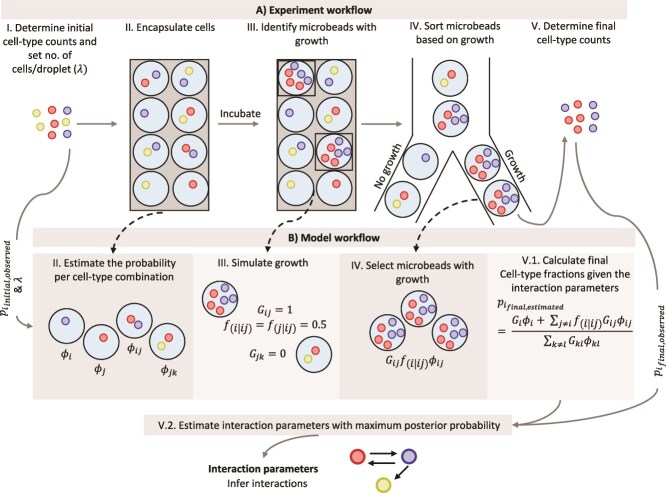
Schematic overview of the experimental workflow (A) and the corresponding modelling steps (B) to decipher pairwise interactions in a microbial consortium. We first determine the abundances of individual cell types in a microbial consortium (I.A) and then encapsulate cells in microbeads at a pre-determined Poisson rate $\lambda$ i.e. at an expected number of cells per microbead (I.B). Using the initial abundances of cell types and the $\lambda$ – value, we can estimate the probability of each cell type combinations in the microbeads (${\varnothing}_{ij}$) (II.B). After incubating until the stationary phase, we identify microbeads with growth using flow cytometry (III.A). In the model, growth in microbeads can be simulated using interaction terms; growth terms (${G}_{ij}$) and strain-fraction term (${f}_{i\mid ij}$) (III.B). Microbeads with growth are sorted (IV.A, IV.B), and the final abundances of cell types are determined (V.A). Using the $\lambda$ – value, interaction parameters and initial and final cell-type fractions, we can calculate the final cell-type fractions (V.B.1). We estimate interaction parameters with the maximum posterior probability by incorporating potential prior information on the interaction parameters and the final cell type counts (V.B.2). Using these interaction parameters, we can then infer interactions between the consortium members.

We developed a label-free, isolate-independent method to infer microbial interactions in consortia (Fig. 1), enabling analysis without the need for genetic engineering or prior isolation of individual consortium members. The approach also does not require prior knowledge of the nutritional requirements of the consortium members. It only relies on cell concentration and relative abundance data under the tested conditions. The approach consists of three key steps:

#### High-throughput cultivation of sub-consortia

Consortium members are randomly encapsulated in oil-separated microbeads, each acting as an independent growth compartment. This allows cultivation in ~10^7^ microbeads (40 μm diameter) per ml. The distribution of cells is inferred from Poisson and multinomial distribution, which are determined by the total inoculum cell concentration and composition. Because cells are partitioned over a large number of compartments (~10^7^ per sample), stochastic deviations from the expected fractions are averaged out.

#### Growth-based separation and composition analysis

We use an autofluorescence signal threshold to distinguish and sort sub-consortia that show growth. This is based on a shift in the autofluorescence signal of microbeads with growth of cells compared to microbeads that were only inoculated (Fig. S5). The relative abundances of cell types are then determined in the sorted population (Fig. 1A).

#### Inference of interaction parameters

We use a simple interaction model together with a Bayesian inference procedure to estimate interaction parameters for all cell-type pairs present in the consortium. We explicitly model the multinomial sampling that leads to the observed cell-type counts, incorporate prior knowledge where applicable, and report uncertainty estimates on all inferred values (Fig. 1B).

### Synthetic consortia design for method validation

**Figure 2:**
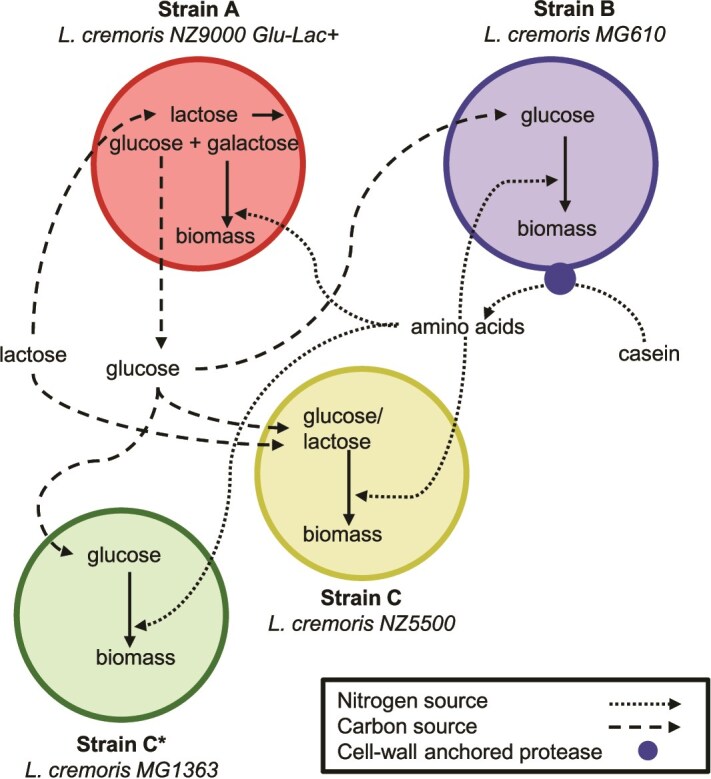
Synthetic consortia of *L. cremoris* strains used in this study. Strain A uses the galactose moiety of lactose as a carbon source and secretes glucose. It uses peptides (amino acids) as a nitrogen source. Strain B uses glucose as a carbon source. It can use casein as a nitrogen source by degrading it extracellularly into peptides. Strain C uses lactose and glucose as carbon sources and peptides as a nitrogen source. Strain C^*^ uses only glucose as a carbon source and peptides as a nitrogen source.

To test our approach, we used two synthetic consortia of *L. cremoris* strains with known interactions. *L. cremoris* can grow both in the presence and in the absence of oxygen, and it can reach high cell concentrations in microbeads. Both consortia contain the same bi-directional cross-feeding strains, A and B (Fig. 2). Strain A (*L. cremoris* NZ9000Glu-Lac+) hydrolyses lactose intracellularly, uses galactose, and secretes glucose; it relies on peptides for nitrogen [10]. Strain B (*L. cremoris* MG610) uses glucose and casein as carbon and nitrogen sources, respectively [11]. It expresses an extracellular protease that hydrolyses casein into peptides. The two consortia differ in the third strain: C or C^*^. Strain C (*L. cremoris* NZ5500) uses both lactose and glucose and peptides [12]. Strain C^*^ (*L. cremoris* MG1363) uses only glucose and peptides [13].

In a medium with lactose and casein as the sole carbon and nitrogen sources, respectively, all strains require at least a partner strain to grow (SI: 3). Growth is ultimately limited by acidification due to lactate production, from now on referred to as growth until medium depletion. Natural systems often consist of many nutrients; this allows low-level background growth of individual species [16–19]. To mimic this, we added low concentrations of glucose and amino acids to the lactose-casein medium (CDMpc_cas,aa,glu + lactose). The data on the growth of the mono- and co-cultures in suspension under conditions that mimic microbead growth, including inoculum cell concentration, medium composition and incubation conditions are provided in Fig S3 and Fig. S4*.* This data is used later in the study to validate the inferred pairwise interactions.

### Determining the growth of individual consortium members

To test whether cells grow individually until medium depletion, we inoculated microbeads such that most filled microbeads contain one cell (at Poisson rate $\lambda$= 0.02, 99% of the filled microbeads contain one cell) (Fig. S2).

As a positive control for growth, we inoculated the consortia (Fig. 2) in CDMpc + glucose + lactose, in which all cells can fully grow. After incubation, we analysed the emulsions by flow-cytometry and, as expected, we observed a shift in the fluorescence signal for microbeads with growth compared to a sample of empty microbeads (Fig. S6). In contrast, when the consortia were inoculated in microbeads of CDMpc_cas,aa,glu + lactose medium, as expected, we did not observe a shift in the fluorescence signal for microbeads with growth compared to a sample of empty microbeads (Fig. S6).

Altogether, these results indicate that the consortium members (Fig. 2) individually do not grow in CDMpc_cas,aa,glu + lactose medium, which is consistent with the results we observed in suspension cultures (Fig. S3).

### Deciphering pairwise interactions

**Figure 3:**
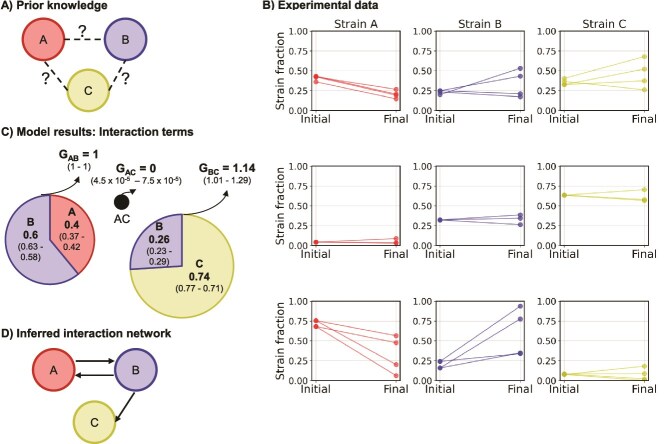
Inference of all pairwise interactions in a three-member consortium. (A) Prior knowledge: We provide no prior information on the interaction parameters. (B) Experimental data depicting the fractions of strains before and after microbead sorting. Microbeads with CDMpc_cas,aa,glu + 0.5 wt% lactose medium were inoculated at a Poisson rate $\lambda$ of 0.3. Each panel represents data from experiment sets with different initial strain fractions. Individual lines represent individual experimental replicates (n = 3 or 4). Strains A, B, and C are represented by red, blue, and yellow colours, respectively. (C) Interaction parameters predicted by the probabilistic model. Each circle represents a pairwise interaction, and the radius of the circle is proportional to the growth of strain pairs relative to the growth of strain pair A and B. The slices of the circles represent the proportions of the individual strains in a pair after growth. The posterior probability interval of the inferred parameters is within brackets. (D) Inferred pairwise interactions from the interaction parameters.

To test whether our method can resolve pairwise interactions, we applied it to a consortium of strains A, B, and C (Figs 2 and 3) and evaluated whether we could predict the three pairwise interactions. We prepared three sets of emulsions; each inoculated with a different set of randomly chosen initial abundances of strains (Fig. 3B). The random selection of initial strain abundances was based on the rationale that inference of interaction parameters is independent of the starting composition, provided that all strains are represented. The microbeads prepared with the medium CDMpc_cas,aa,glu + lactose were inoculated such that most of the filled microbeads contain no more than 2 cells per microbead (Fig. S2) ($\lambda$= 0.3, 90.25% of microbeads with two or more cells contain two cells). By using this Poisson rate $\lambda$, we maximise microbeads with two cells and minimise microbeads with three or more cells. After estimating initial abundances of strains, we inoculated, incubated, and sorted microbeads with growth. Finally, we determined the final abundances of strains.

Using the data on the growth of monocultures, initial abundances of strains (Fig. 3B), the Poisson rate $\lambda$, and the final abundances of strains (Fig. 3B), we employed our Bayesian inference model to estimate the interaction parameters for the three pairwise interactions (Fig. 3C & SI: 6).

Since we can only predict relative growth between strain combinations, we fix the growth of the strain pair A and B to 1. The proportions of strains A (0.40, posterior probability interval or ppi 0.37–0.42) and B (0.60, ppi 0.58–0.63) after growth and the absence of monoculture growth of the strains until medium depletion, suggest a bi-directional cross-feeding interaction between the strains (Fig. 3C and D). This conclusion is supported by suspension culture data, which shows growth of the strain pair until medium depletion and similar strain proportions after growth (Fig. S3). This interaction is attributed to cross-feeding, where strain A provides glucose and strain B supplies amino acids (Fig. 2).

The growth terms of the strain pair B and C (1.14, ppi 1.01–1.29), the proportions of strains B (0.26, ppi 0.23–0.29) and C (0.74, ppi 0.71–0.77) and the absence of monoculture growth of the strains until medium depletion, suggests that strain B facilitates the growth of strain C (Fig. 3C and D). This interaction is further supported by data from suspension cultures (Fig. S3). The growth of strain B is limited by the small amount of glucose in the medium, and strain C also competes for the glucose. Assuming both strains exhibit equal growth rates and cell yields on glucose, the initial “background” growth of each strain can be estimated to 2 cells per microbead, and we expect the final total cell concentration of nine cells per microbead. Based on this, we expect the final proportion of B to be ~0.22, which aligns with the model predictions. This interaction could be attributed to strain B’s ability to grow on the limited glucose, producing sufficient amino acids to support strain C’s growth on lactose until medium limitation (Fig. S3).

The growth term for strain pair A and C (4.5 *·* 10^−5^, ppi 4.5 *·* 10^−5^ – 7.5 *·* 10^−5^) suggests that they do not grow when co-localised (Fig. 3C and D). These results correspond to what we observed in suspension cultures (Fig. S3).

Altogether, we demonstrate that using microbead sorting, we can decipher all the pairwise interactions within a three-member consortium. Despite not providing any prior information to the inference model, it accurately predicts interaction parameters. Moreover, the accuracy of the inferred parameters indicates that our assumed microbeads occupancy in the model closely matched the true occupancy, similar to what was observed in previous studies [7, 19].

### Extending method validation to a different consortium using prior information

**Figure 4:**
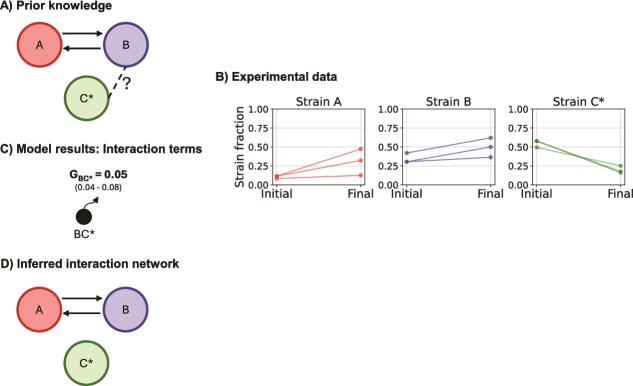
Inference of an unknown pairwise interaction in a three-member consortium using prior information. (A) Prior knowledge where prior information for interactions between strain pairs A-B and A-C^*^ is provided, and no prior information is provided on the interaction parameters for strain pairs B-C^*^. (B) Experimental data depicting the change in fractions of strains before and after microbead sorting. Microbeads with CDMpc_cas,aa,glu + 0.5 wt% lactose medium were inoculated at a Poisson rate $\lambda$ of 0.3. Each panel represents data from experiment sets with different consortia. Individual lines represent individual experimental replicates (n = 3). Strains A, B, and C^*^ are represented by red, blue, and green colours, respectively. (C) Interaction parameters for the interactions between strains B and C^*^ predicted by the probabilistic model. Interactions between strains A and B and strains A and C^*^ are fixed. Each circle represents a pairwise interaction; the radius of the circles is proportional to the growth of the strain pairs and B and C^*^. The slices of the circles represent the proportions of the individual strains after growth. The posterior probability interval of the inferred parameters is within brackets. (D) Inferred pairwise interactions between strains B and C^*^ from the interaction parameters.

To further validate the approach, we tested whether we could decipher a pairwise interaction in another consortium (Fig. 4). The consortia in Fig. 2 have the same strains A and B but differ in strain C^*^. We tested whether we could decipher the interaction between strains B and C^*^. We therefore repeated the same experiment with the consortium A, B, and C^*^ with the goal to infer the interaction parameters between the strain pair B-C^*^. We used our Bayesian model by incorporating prior information on the known interaction parameters; we thus set the interaction parameters to the parameters predicted in the previous experiment (Fig. 3C).

The growth term for the strain pair B and C^*^ (0.05, ppi 0.04–0.08) indicates that these strains, when co-localised, do not grow (Fig. 4C). This result corresponds to what we observed in suspension cultures and to our knowledge of the synthetic consortium (Fig. S3). Although strain B’s growth on the limiting amount of glucose might be sufficient to support the growth of strain C until medium depletion by using lactose as the carbon source, strain C^*^ cannot use lactose and grow until medium depletion despite the presence of sufficient amounts of amino acids.

Altogether, these results support that we can reliably decipher pairwise interactions in two different consortia. Furthermore, the accurate prediction of interaction parameters using prior information from a previous experiment with a similar consortium demonstrates the iterative nature of the approach.

### Computationally deciphering pairwise interactions in larger consortia

We aimed to computationally evaluate whether we can decipher pairwise interactions within larger consortia; we designed consortia ranging from 4 to 6 members. Like in the experiments, we chose a Poisson rate $\lambda$ of 0.3 to maximise microbeads with two cells and minimise microbeads with three or more cells i.e. at this $\lambda$ 90.2% of microbeads with two or more cells contain two cells.

We simulated the growth of the consortia at for different numbers of experiments, where each experiment is initiated with a random set of initial cell-type proportions in the consortium. Each experiment provides us with measurements of the initial and final cell-type proportions; in a consortium with $N$cell types, we get $N-1$independent measurements. However, for these $N$cell types, we need to fit $\frac{\left(N-1\right)N}{2}$ growth factors ${G}_{ij}$, and $\frac{\left(N-1\right)N}{2}$ relative proportions ${f}_{i\mid ij}$. We therefore expect better prediction of interaction parameters for larger consortia by the inference model as the number of experiments and thereby the number of independent measurements increases. Particularly when the number of independent measurements exceeds the number of unknown interaction parameters.

**Figure 5:**
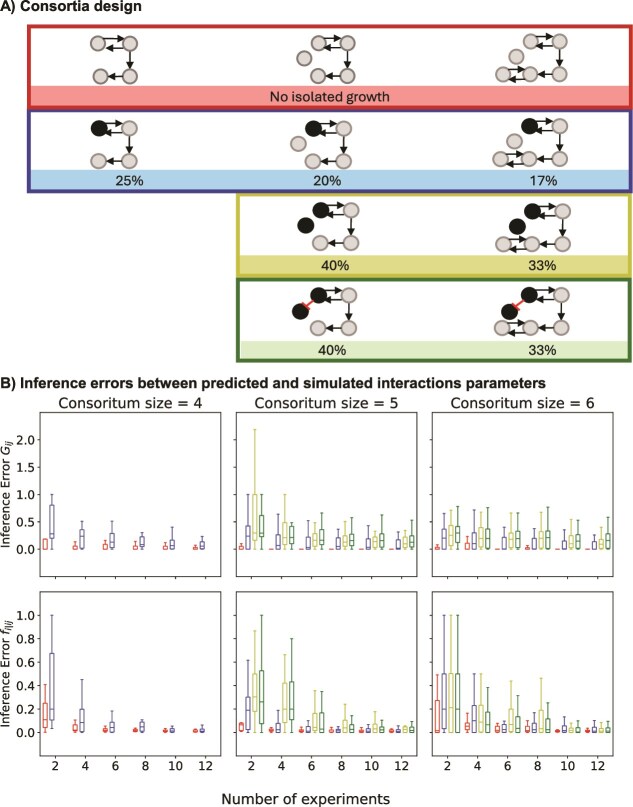
Computational validation to infer interactions in larger consortia, “low noise” conditions. Inference errors between predicted and simulated interaction parameters. (A) Consortia were designed for simulation; the cell types that do not grow by themselves, i.e. with no isolated growth, are represented in grey and cell types that grow by themselves are represented in black. The percentage of consortium members capable of isolated growth is indicated below each consortium. (B) The distribution of inference errors of the growth (${G}_{ij}$) and cell-type fraction terms (${f}_{i\mid ij}$) for different numbers of experiments, where the inference error is the absolute difference between the interaction parameters predicted by the inference model and the corresponding interaction parameters used in the simulation. For these simulations the Poisson rate $\lambda$ was set to 0.3 and number of colonies to determine cell type abundances was set to 1000. The number of experiments corresponds to the number of unique sets of initial cell-type counts. The individual box plots represent the distribution of the inference errors for five random replicates for the same number of experiments. The colours of the box plots correspond to the consortia configurations in panel (A).

Based on five sets of random initial fractions of consortium members (i.e. five replicate simulated datasets) and the consortia design, the final fractions were calculated with the simulation model (SI:7). We ignore all higher-order interactions (interactions between three or more cell types) in the simulations as well as in the inference model, since we set the Poisson rate $\lambda$ to 0.3, where only 1% of microbeads with cells contain three or more cells. We estimated inference errors per interaction parameter—absolute differences between the interaction parameters predicted by the inference model and the corresponding interaction parameters used in the simulation. We estimated two sets of inference errors: one for the growth term (${G}_{ij}$) and one for the cell-type fractions (${f}_{i\mid ij}$) (only considered when ${G}_{ij}$ > 0.1). We represented these inference errors with the distribution from the five random sets of initial fractions (independent replicates) (Fig. 5).

We simulated sampling either 96 colonies or 1000 colonies to estimate the abundances of the different cell-types for the inference model. In our Bayesian inference model, we only explicitly model the noise from multinomial sampling when estimating cell-type counts, assuming the other experimental sources of noise are negligible. Sampling 96 colonies (Fig. S9) represent a “high noise” scenario, and 1000 colonies corresponds to a “low noise scenario” (Fig. 5). The decrease in the average inference error with an increased number of sampled colonies could be attributed to decreased sampling noise. Therefore, in the analyses below, we focus on the 1000 colonies sampling condition, where lower noise levels allow clearer visualisation of the observed trends.

In our simulations, we constructed consortia in which none of the cell types could grow independently (Fig. 5A). In this case, we observed that the inference errors are low regardless of the consortium size (Fig. 5B). We then incorporated one or two independently growing cell types into the simulated consortia (Fig. 5A); we define this as isolated growth. In the presence of isolated growth, for a given consortium size, we observed that the inference errors in most cases increase with the proportion of cell types capable of isolated growth (Fig. 5B). For example, irrespective of consortium size and the number of experiments, inference errors for both interaction parameters are significantly higher (*P* < .05) for consortia with one cell type capable of isolated growth compared to consortia with no isolated growth. In addition, for consortia of sizes 5 and 6 across different number of experiments, inference errors for both interaction parameters are significantly higher (*P* < .05) in consortia with two cell-types capable of isolated growth compared to those with only one such cell type. While the inference errors generally decrease with an increasing number of experiments, they do not reach the lower errors observed in conditions without isolated growth (Fig. 5). This is expected since, given that the expected number of cells per microbead is 0.3 in these simulations, many of the microbeads with cells (=86%) will have only 1 cell. As a consequence, when cell types can grow in isolation, they will heavily contribute to the final population of cells. Although larger errors are expected, the model can still recover the interaction parameters to a reasonable accuracy. In addition to incorporating isolated growth, we introduced a negative interaction between two cell types capable of isolated growth (Fig. 5A). The resulting inference errors followed the same trend as that observed in the corresponding consortia with isolated growth alone (Fig. 5B). The errors were not significantly different (*P* > .05). In summary, the inference errors are lowest under conditions without isolated growth and increase with the rise in the proportion of consortium members capable of isolated growth for a given consortium size. Introducing a negative interaction between cell types capable of isolated growth does not alter this trend.

We validated the method for a range of interactions, including commensalism (+/0), mutualism (+/+), and amensalism (0/−). The same approach can be extended to decipher other negative interactions, such as competition (−/−) and antagonism (+/−), as well as neutral interactions (0/0). Using this framework, we can infer these interactions based on whether a cell-type’s growth decreases (negative) or remains unchanged (neutral) when co-localized with another cell-type.

## Discussion

We developed a novel microbead-based approach to decipher microbial interactions in a label-free, isolation-independent manner. Unlike existing microbead-based methods that require single culture isolates and the expression of fluorescent markers via genetic engineering [7–9], our approach enables inoculation of microbeads with all possible sub-consortia combinations and enrichment of sub-consortia based on growth, using autofluorescence as an indicator. An additional advantage of this separation of sub-consortia is that slow-growing species can be spatially separated from fast-growing ones, thereby reducing the influence of the fast growers on the final abundance data. We validated this approach by deciphering pairwise interactions in two synthetic three-member consortia.

Natural microbial communities are often found in nutrient-rich environments, such as the gut. In such environments, we expect background growth of consortium members, i.e. individual growth to a low cell concentration. However, in the presence of another consortium member with a positive interaction (such as commensalism and mutualism), a higher cell concentration is reached [16–19]. Background growth adds noise to the relative abundance data, making it challenging to derive interaction parameters. This was shown in a study where sub-consortia in microdroplets were intermittently mixed between each propagation [19]. In the early rounds, background growth of monocultures introduced noise into the relative abundance data, complicating accurate identification of interactions. As a result, multiple rounds of serial propagations were required to obtain reliable interaction data. To reduce this noise in the data, we incorporated microbead sorting. Using microbead sorting, we can select monocultures and sub-consortia that grow until medium depletion and eliminate the microbeads with only background growth. Our results show that we can decipher pairwise interactions among the consortium members despite the presence of background growth.

We demonstrated computationally that interactions can also be deciphered in larger consortia. The prediction accuracy was highest without isolated growth. For a given consortium size, the accuracy decreased as the proportion of cell types capable of isolated growth increased. More experiments, i.e. providing more data, improved the prediction accuracy, but errors remained higher than in cases without isolated growth. This pattern persisted even in the presence of a negative interaction, such as amensalism, because, at a Poisson rate $\lambda$ of 0.3, 86% of microbeads inoculated with cells contain only one cell. This results in a high relative contribution of cell types capable of isolated growth to changes in the final cell-type fractions, reducing data resolution for accurate interaction parameter inference. Increasing $\lambda$ may improve the inference accuracy by reducing the proportion of microbeads inoculated with a single cell. However, this would increase microbeads with three or more cells, introducing higher-order interactions (where the presence of a third community member modifies a pairwise interaction) that require additional model parameters. Alternatively, controlled inoculation of all microbeads with two cell types per microbead using surface acoustic waves or inertial focusing [20, 21], could improve inference.

Although we showcase our method using synthetic microbial consortia, it has the potential to be adapted to natural microbial communities. This additionally requires (i) stabilising consortia isolated from environmental samples, (ii) determining relative abundances of cell types in both the inoculum and post-sorting, and (iii) generating inocula with varied relative abundance profiles. Stabilisation can be achieved by serial passaging in the target medium to filter out non-growing cell types [22, 23]. Relative abundances can be accessed via amplicon sequencing of species-specific rRNA genes [1]. To introduce variations in relative abundances, without isolating individual species, we can introduce stochastic methods such as serial dilution with random aliquoting of different dilutions, though this may favour dominant species, or use perturbations like nutrient pulses, temperature shifts, or pH changes.

The application of the proposed approach in this study to enable inference of microbial interactions directly from natural communities still faces several limitations. First, the method relies on autofluorescence to detect growth in sub-communities, but autofluorescence could vary across species due to physiological differences. We can overcome this by using alternatives, such as staining for viability [24] or metabolic activity [9], or tracking droplet-level changes in size [25] or weight due to growth [26]. Second, species-specific traits can also introduce bias. For instance, agarose-degrading microbes may break down microbeads, causing loss of microbeads harbouring these cell types and skewing the estimation of interaction parameters. We can mitigate this by switching to alginate microbeads or adopting water-in-oil-in-water emulsions, which are compatible with microbead sorting. A key limitation, however, is that the method currently is restricted to soluble carbon sources and nutrients that do not readily partition into the oil phase [27].

Commonly used ecological models like the Generalised Lotka-Volterra model and Generalised Consumer-Resource model define interactions by employing growth rates and abundance data over time [4, 6, 28]. Our approach does not consider growth rates but defines interactions based on abundance data from a single time point, usually after medium depletion. This limits our approach in capturing dynamic changes such as growth-dependent interactions and cell-concentration-dependent co-operation [29]. However, after microbead sorting, each enriched sub-consortia can be characterised further in suspension cultures to track dynamic changes. Thus, our approach acts as an initial high-throughput screen, after which sub-communities of interest can be investigated further with more specific methods.

In addition to determining pairwise interactions, our method has the potential to decipher higher-order interactions. The challenge with determining higher-order interactions is the additional interaction parameters to estimate. To solve this problem, we envision an iterative scheme. We first inoculate at a low Poisson rate $\lambda$ to determine monoculture growth parameters. By inoculating with a low number of cells per microbead, we ensure that cells are growing by themselves, such that these growth parameters can be inferred reliably. Subsequently, we can increase the Poisson rate $\lambda$ to infer pairwise interactions. In this iteration, we can then use the previous results on the monoculture growth parameters to constrain the prior expectation for these parameters. Knowing the pairwise interactions, we can proceed to determine triplet interactions. To have sufficient resolution to predict triplet interactions, it is crucial to balance the proportion of microbeads inoculated with three or more cells relative to those that show growth when inoculated with one to two cells. Determining higher-order interactions using microbead-based methods is also constrained by the carrying capacity microbeads. If the carrying capacity of a microbead is 10 cells, then we are limited to deciphering pairwise interactions to ensure sufficient cell doublings within a microbead. We can mitigate the carrying capacity limitation by alleviating medium limitation (sugar, pH, nutrients, etc.) and/or by increasing the bead size. However, for droplets or beads larger than ~50 pL (45 μm), conventional FACS is no longer suitable; instead, we require large particle sorters [30].

In light of future experimental developments, it is important to note that the Bayesian inference model developed in this study explicitly only models the multinomial sampling noise arising from our plating-based determination of the initial and final cell-type frequencies. However, we ignore other potential noise sources (such as errors introduced during microbead-sorting, or biological variation in growth between cells from the same strain in different microbeads, contribution of higher order interactions). Under the current experimental conditions, these noise sources were negligible compared to the multinomial sampling noise. This assumption may not hold in slightly altered experiments, e.g. when working with larger consortium sizes or less well-characterized strains. Therefore, itis essential to identify the dominant sources of measurement noise and adapt the model accordingly.

Although alternative microdroplet-based methods enable inference of higher-order interactions and control over microbead co-occupancy, they typically rely on genetically engineering fluorescent labels into each cell type to monitor sub-consortium growth [7–9], which is often not feasible for natural consortia. Species-specific fluorescently labelled rRNA probes (FISH) offer a non-genetic-engineering alternative [1], but traditional FISH compromises cell viability. Promising advances in LIVE-FISH [31, 32] combined with microbead-based cultivation [7–9] could enhance the throughput of deciphering interactions in natural microbial consortia. Apart from deciphering interactions, our method can be applied to elucidate rare growth-promoting interactions which are expected to evolve under restrictive conditions, such as mutualistic cooperation [3, 8, 21, 29]. Existing approaches are limited by the reliance on culturable isolates, which constrains them to a few phylogenetic groups. Since, e.g. cooperation often results in higher cell concentrations, our method could be adapted for high-throughput screening of consortia of such interactions. This could enable the elucidation of interactions yielding higher cell concentrations across diverse environments that reveal different interactions, helping to derive principles driving their evolution and selection.

Our approach can also be extended for developing synthetic consortia for industrial biotechnology applications for recent research on valorising complex mixed substrates from waste streams shows that consortia of specialists—where each member is specialised to convert one of the substrates to the product—outperform generalist microorganism that converts all the substrates to a product [33–35]. However, the key challenge is the stability of these specialist consortia. Since efficient substrate consumption by stable consortia would result in higher biomass yields, our method provides a high-throughput approach to screen for high biomass yields of sub-consortia on substrate mixtures. Thereby, enabling enrichment of stable specialist sub-consortia from a large mutant pool. Beyond growth, the method can be adapted to screen for diverse phenotypes by incorporating stains or assays targeting metabolic activity, respiration, nucleic acid content, and other biochemical traits.

The label-free and isolation-independent approach proposed in this study has the potential to decipher interactions in diverse and unexplored natural microbial consortia. It paves the way for research to understand interactions driving the stability and function of microbial consortia and offers opportunities to define design principles for industrial applications of microbial consortia.

## Supplementary Material

Supplementary_information_ycag058

## Data statement

All data generated or analysed during this study are included in this published article and its supplementary information files.
