# Supplementary material for: Deciphering microbial interactions using a label-free microbead sorting approach
Source: ISME Commun. 2026 Mar 13;6(1):ycag058. doi: 10.1093/ismeco/ycag058 (PMC13064671; doi:10.1093/ismeco/ycag058)
Supplement: Supplementary_information_ycag058 [file supplementary_information_ycag058.pdf]

Supplementary information

Deciphering Microbial Interactions Using a Label-Free Microbead Sorting Approach

Short title: Deciphering Microbial Interactions

Authors: Sagarika B Govindaraju[1], Daan H de Groot[2] and Rinke J van Tatenhove-Pel[1]

[1]Department of Biotechnology, Faculty of Applied Sciences, TU Delft, Delft, The Netherlands.

[2]Department of Computational Systems Biology, Biozentrum, University of Basel, Basel, Switzerland.

Please address correspondence to Rinke van Tatenhove-Pel, R.J.vanTatenhove-Pel@tudelft.nl.

Mailing address for correspondence:

Rinke van Tatenhove-Pel

Department of Biotechnology,

Faculty of Applied Sciences,

Van der Maasweg 9,

2629 HZ Delft, The Netherlands.

SI: 1. Microbead size and volume distributions

We prepared agarose microbead emulsions as described in the materials and methods section. We imaged the resulting polydisperse microbeads with a microscope (Figure S1A, 9 images per emulsion). The images were subsequently analysed with ImageJ to identify the microbeads and determine their size (Figure S1B). Microbeads on the edges of the images and small microbeads (up to 2 μm in diameter) were excluded from the analysis. Based on these ImageJ results, average microbead diameter and volume distributions were determined.

The average number of cells in a microbead is defined by the Poisson rate λ. The Poisson rate λ is dependent on the number of cells added to the water phase and the average size of microbeads. Since we use polydisperse emulsions, we have a distribution of microbead sizes. Although smaller microbeads (< 20 μm) are more abundant (FigureS1D), due to a low volume of these microbeads, they occupy a small fraction of the total water phase (FigureS1C). When cells are homogenously mixed in the water phase, only a small fraction of the cells is harboured within the small microbeads due to the low volume fraction of the water phase occupied by these microbeads. Most of the cells in the water phase will be distributed over the larger microbeads. We reasoned that the average diameter of microbeads that occupy the highest volume fraction represents the average diameter of the emulsion and can be used to determine the Poisson rate λ.

The average diameter (± standard error of the mean) of microbeads that occupy the largest volume fraction was calculated to be 49.65 pL ± 8.02 pL (44.15 μm ±2.37 μm, n=8).

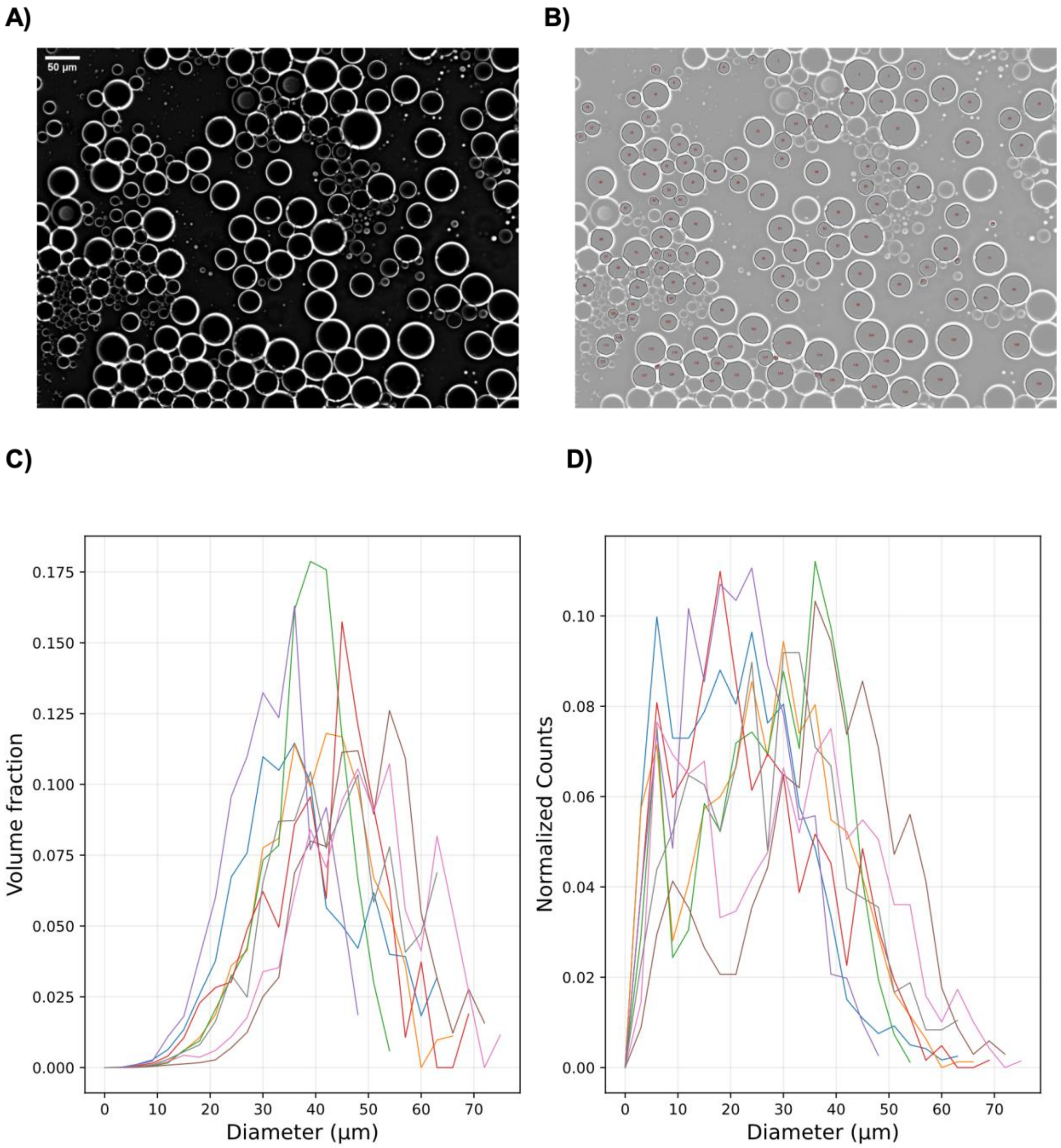

FigureS1: (A) Microscopic image of agarose microbeads. (B) Microbeads identified in (A) by ImageJ analysis. (C) Microbeads diameter distribution based on volume fraction of the water phase and (D) Microbeads diameter distribution based on counts, where counts were normalised to the total number of microbeads analysed per emulsion.

SI: 2. Poisson distribution

To determine the growth of individual consortium members we used a Poisson rate λ of 0.02, at which 99% of filled microbeads contain one cell. However, at this λ, 98% of microbeads are empty. Since we can generate many microbeads (10^7), this still allows us to analyse 10^5 microbeads. (Figure S2)

To determine pairwise interactions, we used a Poisson rate λ of 0.3. At this λ-value, we expect 74% empty microbeads. However, 90% of microbeads with two or more cells contain two cells. This allows us to maximize the number of microbeads with two cells and minimise the number of microbeads with three or more cells.

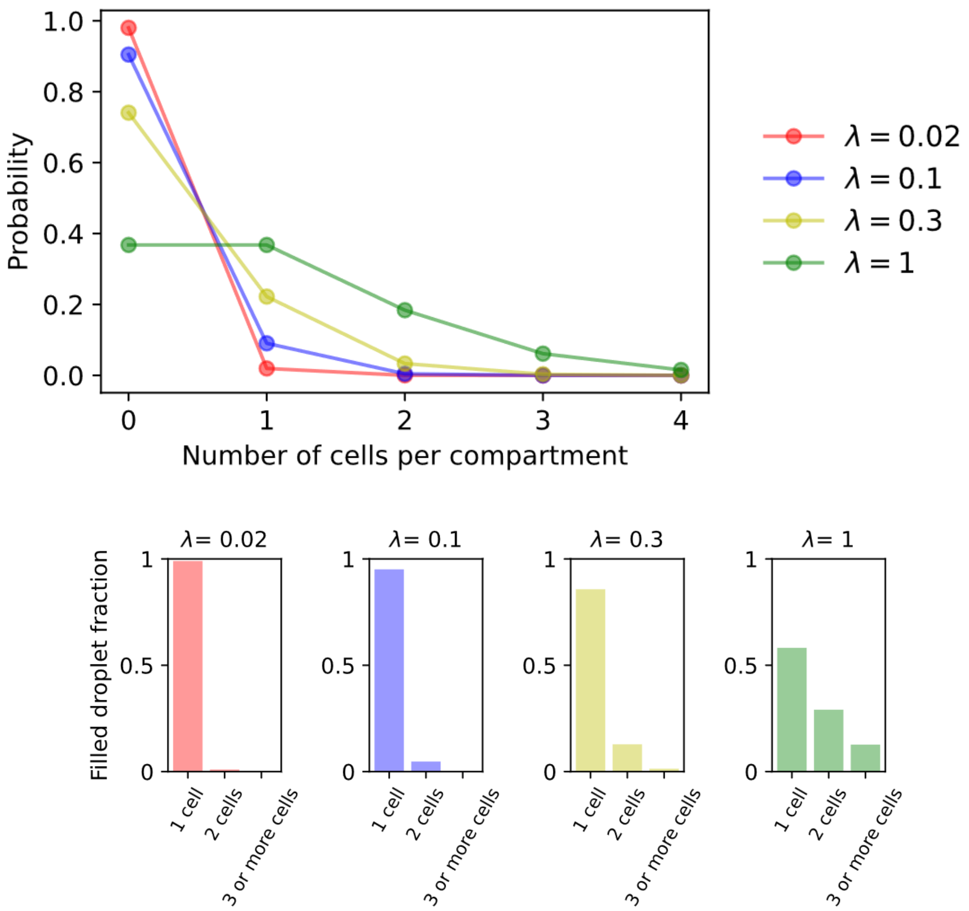

FigureS2: Poisson distribution for Poisson rates λ-values of 0.02, 0.1, 0.3 and 1. The line plot shows the probability of compartments containing 0 to 4 cells per compartment. The bar plot shows the fraction of filled compartments containing 1, 2 and 3 or more cells.

Poisson distribution formula to estimate the amount of cell culture required to prepare emulsions:

V = volume of the water phase (μL, usually 300 μL)

D = mean microbead diameter (μm)

C_{cells} = cell-culture concentration (cells/μL)

λ = average microbead occupancy

A = amount of cell culture to use (μL)

$$A = \frac{\lambda \cdot V \cdot 10^9}{\frac{4}{3} \cdot \pi \cdot (0.5 \cdot D)^3 \cdot C_{cells}}$$

SI: 3. Mono- and co-culture analysis

To test if the consortia grow as expected, we determined the growth of monocultures and co-cultures of the consortia in suspension cultures. These cultures were inoculated in CDMpc_cas,aa,glu + lactose medium at cell concentrations mimicking 2 cells per microbead. Monocultures of strains A and C, limited by amino acids in the medium, exhibited a 1.63 ± 0.047 and 1.86 ± 0.047 fold increase in cell concentrations, respectively (FigureS3). Monocultures of B and C*, limited by glucose in the medium, showed a 2.33 ± 0.26 and 1.73 ± 0.094 fold increase in cell concentrations (Figure S3&S4). In co-cultures, cross-feeding between strains A and B allowed growth until medium depletion, with a 5.03 ± 0.54 fold change in cell concentration (Figure S3). After growth, the proportions of strains A and B were 0.49 ± 0.085 and 0.51 ± 0.085, respectively (Figure S3). The strain pair B and C also grew until medium depletion, with a 5.00 ± 0.24 fold increase in cell concentration (Figure S3). However, only strain C is detected after growth (Figure S3). This suggests that the glucose-limited growth of strain B was sufficient to break down enough casein to supply amino acids for strain C to grow until medium depletion, which uses lactose as the carbon source. Growth of the strain pair A and C was amino-acid limited, with a 1.80 ± 0.082 fold increase in cell concentration (Figure S3). Growth of the strain pair A and C*, limited by amino acids, showed a 2.00 ± 0.082 fold increase in cell concentration (FigureS3). Growth of the strain pair B and C* was limited by glucose, with a 2.06 ± 0.094 fold increase in cell concentration (FigureS3).

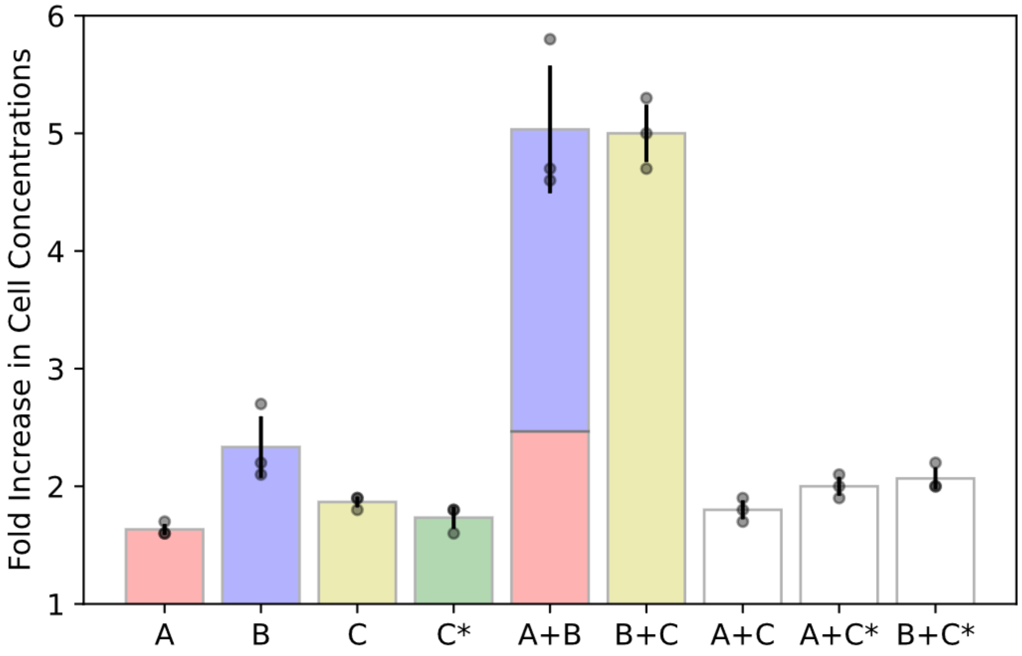

Figure S3: Average fold increase in stationary phase cell concentrations (n=3) of suspension cultures incubated in medium CDMpc_cas,aa,glu + 0.5 wt% lactose for mono-cultures and bi-cultures. Monocultures and co-cultures were inoculated at initial cell concentrations $6{\cdot}10^7$ cells/mL. These inoculation cell concentrations correspond to an average of and 2 cells per 40 μm microbead, respectively. Co-cultures were inoculated with 32.4% A and 67.6% B, 53.6% B and 46.4% C, 54.4% A and 45.6% C, 56.5% A and 43.5% C* and 48.3% B and 51.7% C*. The proportions of strains A, B, C and C* after growth are indicated by the colours red, blue, yellow and green, respectively. Strain pairs A and C, A and C*, and B and C* did not grow; hence, their proportions were not measured after growth.

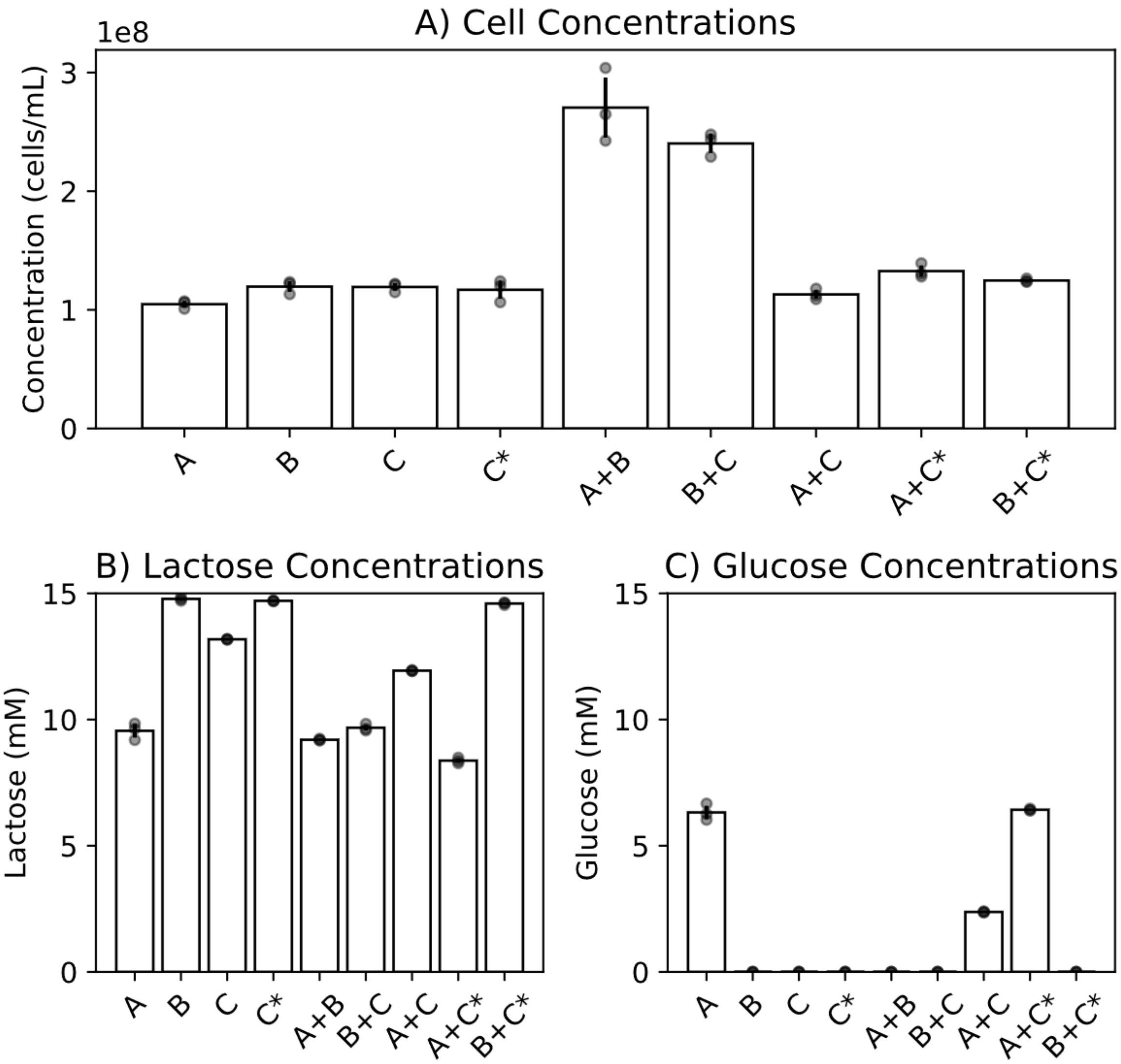

Figure S4: Average cell (A), lactose (B) and glucose (C) concentrations of stationary phase cells (n=3) of suspension cultures incubated in medium CDMpc_cas,aa + 14.6 mM lactose + 1.1 mM glucose for mono-cultures, bi-cultures, and co-cultures. Cultures were inoculated at initial cell concentrations of $6{\cdot}10^7$ cells/mL. These inoculation concentrations correspond to 2 cells per 40 µm microbead. Co-cultures were inoculated at the following ratios: A + B (0.32,0.68), B + C (0.54,0.46), A + C (0.49,0.51), A + C* (0.57,0.43), and B + C* (0.48,0.52).

SI: 4. Assessment of the experimental workflow

To test whether we achieve growth in microbeads, identify microbeads with growth using flow cytometry, and selectively enrich for sub-populations, we designed an experiment with a mixture of wild-type and GFP-expressing *Lactococcus cremoris* strains. We cultured the mixture in microbeads, sorted using a FACS for the high-fluorescence microbead population, and tested if we selectively enriched the GFP-expressing strains from the mixture.

To ensure that each inoculated microbead has only one cell, we inoculated microbeads at a λ-value of 0.1. At this λ-value, 95% of the filled microbeads contain 1 cell per microbead (SI:2). We identified microbeads with growth based on a fluorescence threshold on the FACS (SI: 5, Figure S5). We observed a shift in fluorescence for microbeads with the growth of *L. cremoris* MG1363-GFP and for *L. cremoris* MG1363 (SI: 5, Figure S5). Although *L. cremoris* MG1363 is not fluorescently labelled, we still observed a shift in fluorescence for microbeads with growth of the strain, due to the autofluorescence of the cells. To selectively enrich for *L. cremoris* MG1363-GFP, we sorted microbeads with the highest fluorescence signal, and we subsequently measured the relative abundances of the strains.

The results show that *L. cremoris* MG1363-GFP was enriched from a starting proportion of 7.1 ± 0.4 % to a final proportion of 57.5 ± 13.2 % (n=4). The presence of non-fluorescent *L. cremoris* MG1363 is possibly due to inaccuracies in sorting and possible adhesion of *L. cremoris* MG1363 to the surface of microbeads inoculated with *L. cremoris* MG1363-GFP. Despite these factors, *L. cremoris* MG1363-GFP was enriched by a factor of eight in a single enrichment round.

Altogether, these results show that we can achieve growth of *L. cremoris* strains in microbeads and, using flow cytometry, we can identify microbeads with growth for both fluorescently labelled and unlabelled strains. We also show that we can selectively enrich for a sub-population of cells from a mixture by sorting microbeads using a FACS.

SI: 5. Gating Strategy

Gating was performed using control samples representing individual sub-populations: single cells, empty microbeads, microbeads with growth of fluorescently labelled cells, and microbeads with growth of non-fluorescently labelled cells. (Figure S5)

Note: The presence of single cells populations suggests occasional microbead damage, which we expect to be unbiased i.e., we expect damage to microbeads to be random, not specific to microbeads inoculated with certain strains or combinations. Therefore, this does not affect the inference of interaction parameters. In our experiments, only a small fraction of microbeads (≈1000 beads) out of 10^7 microbeads generated are sorted for parameter inference, so defects in a small fraction of microbeads have negligible impact due to the large number of microbeads available for analysis.

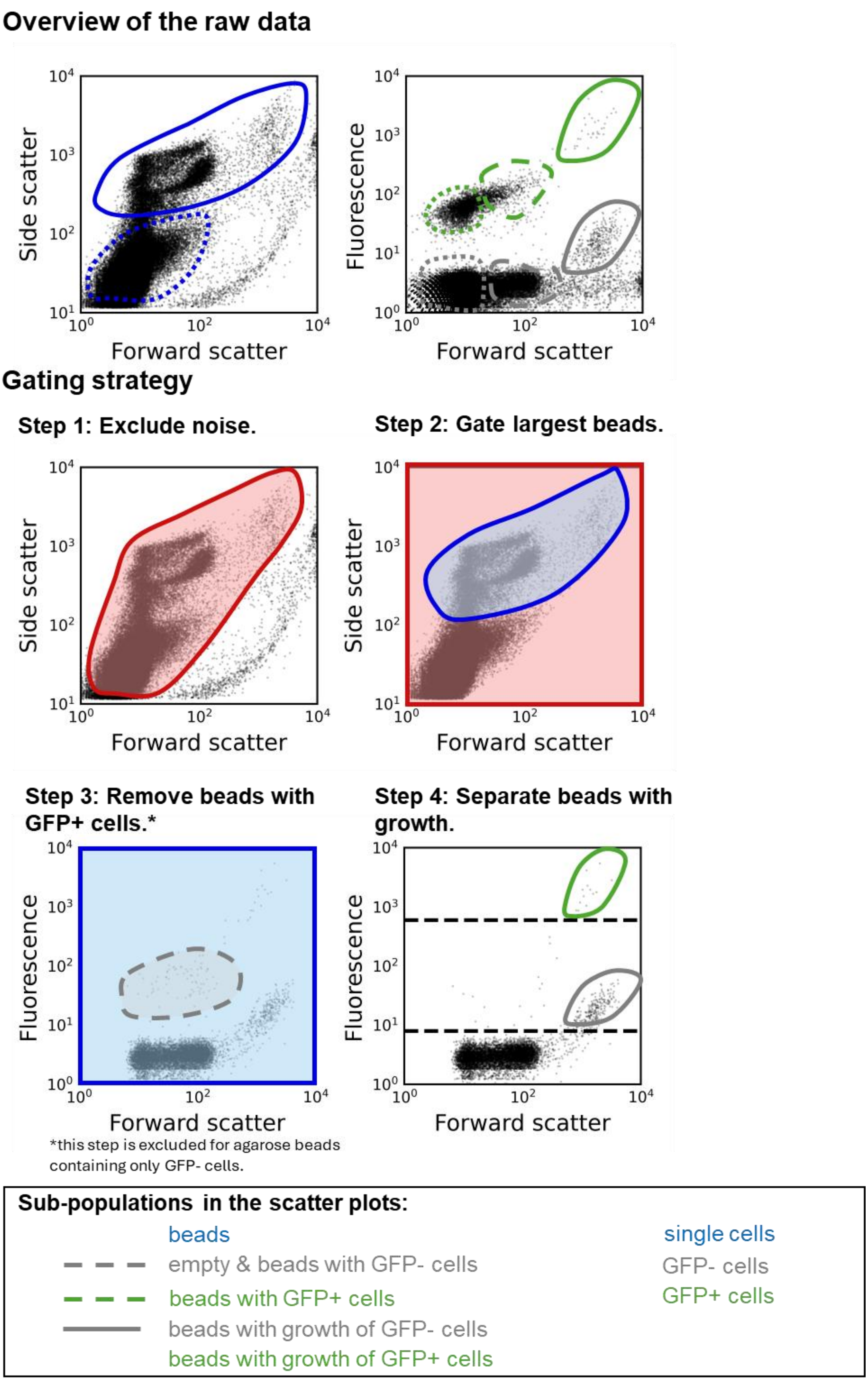

Figure S5: General gating strategy for flow cytometry data.

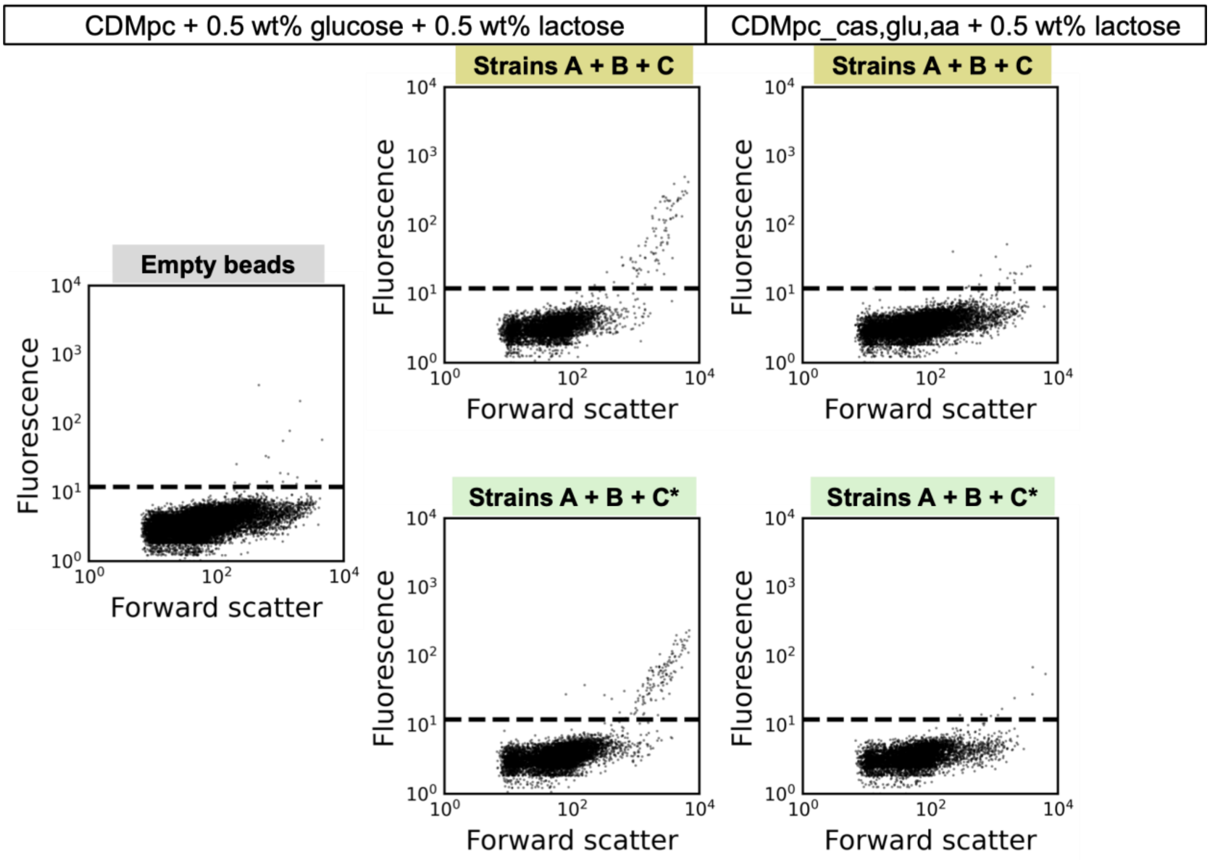

Figure S6: Flow cytometry gating strategy and data analysis. Empty microbeads and microbeads inoculated with a consortium of strains A, B and C (top) and strains A, B and C* (bottom) at a λ-value of 0.02 were measured after incubation. The inoculum consisted of 47% strain A, 29% strain B and 24% strain C (top) or 41% strain A, 21% strain B and 38% strain C*. Microbeads were prepared with medium CDMpc + 0.5 wt% lactose + 0.5 wt% glucose (left) and CDMpc_cas,glu,aa + 0.5 wt% lactose. Flow cytometry data were gated based on the strategy in Figure S8. The scatter plots are representative plots from three independent replicate samples.

SI: 6. Bayesian inference of the interaction parameters

In this section, we describe how we infer the pairwise interaction parameters from the measured cell type abundances. The available data are measurements of the cell-type proportions before and after the growth phase. These proportions are measured by selecting a random number of cells and determining their cell type. For each experiment, we thus get a vector, $\vec{d}(0)$, with cell-type counts **before** the growth phase, and a similar vector, $\vec{d}(T)$, of cell-type counts **after** the growth phase. Here, we will, given a set of parameters $G_{ij}, f_{i|ij}$, write down an expression of the likelihood. By introducing prior distributions on the parameters, we subsequently write down the posterior probability for the parameters. Given this posterior probability function, we numerically find the parameter values that have maximum posterior probability, and a corresponding posterior probability interval.

The likelihood will reflect the different steps in the experiment, which we will describe in order.

Step 1: Determining initial cell-type fractions

Before inoculating the microbeads, the cells are mixed to obtain targeted proportions (captured by a vector $\vec{t}$). However, we can not be certain that the true proportions $\vec{p}(0)$ exactly match the targeted fractions. Therefore, we take a sample of cells and determine their cell type, leading to counts $\vec{d}(0)$. The probability of these counts is multinomially distributed with probabilities given by the true proportions, $\vec{p}(0)$:

$$P(\vec{d}(0).|\vec{p}(0)) = \frac{(\sum_i d_i(0))!}{d_1(0)!\, d_2(0) \cdots} \prod_i p_i(0)^{d_i(0)}$$

The targeted proportions give us prior information on the true fractions, which we capture by using a Dirichlet prior centred around the fractions t and with a precision controlled by a hyperparameter ν:

$$P(\vec{p}(0)) = \Gamma(\nu)\prod_i \frac{p_i(0)^{\nu t_i - 1}}{\Gamma(\nu t_i)}.$$

This Dirichlet distribution peaks around the targeted fractions t_i, and this peak gets steeper when the precision ν increases. By multiplying the probability of the data and the prior, we get an expression proportional to the posterior for $\vec{p}(0)$, which is again taking the shape of a Dirichlet distribution:

$$P(\vec{p}(0)|\vec{d}(0), \vec{t}, \nu) \propto C(\nu, t_i, d_i(0)) \times \prod_i \frac{p_i(0)^{\nu t_i + d_i(0) - 1}}{\Gamma(\nu t_i + d_i(0))},$$

where $C(\nu, t_i, d_i(0))$ is a term that does not depend on $\vec{p}(0)$. Note that this distribution thus gives a full distribution of possible initial fractions. However, we will explain below that the experimental results are best explained by a large value for ν, which is the experimental precision in preparing the strain frequencies in the inoculum. As a result the uncertainty on $\vec{p}(0)$is generally low, so we will ignore it in the following, and just assume that these proportions are equal to their maximum posterior estimate. This is given by:

$$p_i(0) = \frac{\nu t_i + d_i(0)}{\nu + \sum_i d_i(0)}$$

<u>Optimisation of the precision parameter, ν</u>

The parameter ν captures the precision with which the cell-type fractions are prepared. This precision is independent of the experiment, which is why we use several experiments to determine this parameter. We thus determine ν by maximizing the

probability of the data for several experiments. For this, let $P(\overrightarrow{d^e}(0), \overrightarrow{p^e}(0)|\overrightarrow{t^e}, \nu)$be the joint probability of counts and true proportions for the experiment e. The total probability of the data given the parameter ν is given by:

$$P(\overrightarrow{d^1}(0), \dots \overrightarrow{d^E}(0)|\nu) = \prod_e \left(\int d\overrightarrow{p^e}(0) P(\overrightarrow{d^e}(0), \overrightarrow{p^e}(0)|\overrightarrow{t^e}, \nu)\right).$$

Note that we here marginalize over the true fractions $\vec{p}(0)$ because we are only interested in the dependence of the probability of the data on ν. This integral is easy to solve by using that the joint probability is directly proportional to a Dirichlet distribution. We used the end result to numerically maximize the likelihood of ν to get $\nu = 91.66$ (Figure S7).

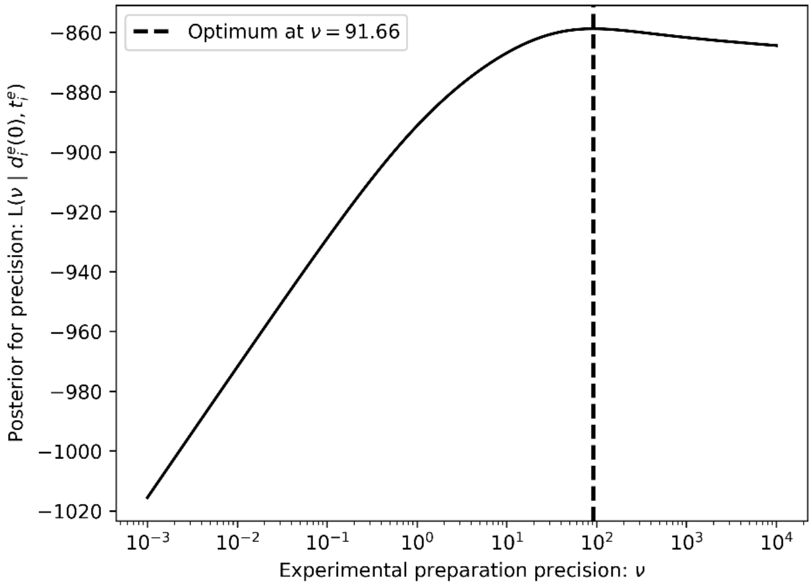

Figure S7: Probability of the initial count data as a function of experimental preparation precision ν. The dashed vertical line indicates the optimum at ν =91.66.

Step 2: Distribution of cells over microbeads

Now that we have estimated the cell-type proportions in the inoculum, we can describe the random distribution over the microbeads. The number of cells per microbead is

Poisson-distributed with a mean λ. The number of cells in a microbead is thus described by:

$$P(n) = \frac{\lambda^{n} e^{-\lambda}}{n!}$$

In this work, we experimentally set the λ to 0.3, which ensures that, although most of the microbeads (=96%) contain 0 or 1 cell, most of the microbeads with 2 or more cells contain 2 cells (=90%). For this reason, in the model, we ignore the small percentage of microbeads with 3 or more cells.

We can now compute the frequencies at which the different cells occur in microbeads. The fraction of microbeads with a pair i and j is given by

$$\phi_{ij} = \frac{\lambda^2 e^{-\lambda}}{2} \times 2p_i(0)p_j(0) = \lambda^2 e^{-\lambda} p_i(0)p_j(0).$$

Here, we thus multiply the probability of getting a microbead with 2 cells given by the Poisson distribution with the probability that these cells are of types i and j. Similarly, the fraction of microbeads with only one cell type i is given by:

$$\phi_i = \lambda e^{-\lambda} p_i(0) + \lambda^2 e^{-\lambda} p_i(0)^2$$

We assume that the fraction of microbeads for a given cell type pair is given exactly by these probabilities since the number of microbeads in a sample is large (~10^7 microbeads).

Step 3: Growth of cells in the microbeads

Cells that are co-localised in a microbead will interact with each other and grow based on the interactions until resources in the medium are depleted. We characterise the growth by two interaction parameters: G_{ij} and $f_{(i|ij)}$. G_{ij} denotes the average number

of cells which will eventually exist in a microbead that was inoculated with an i, j-pair, while $f_{(i|ij)}$ denotes the fraction of the cells in the droplet that will be of type i (Note that we thus have $f_{(i|ij)} = (1 - f_{(j|ij)})$. For a microbead inoculated with an i, j-pair, the eventual number of cells of type i will thus be $G_{ij} \times f_{(i|ij)}$. Therefore, the final number of cells of type i can be expressed in terms of these parameters and the probabilities of finding a certain pair of cell-types in a microbead ϕ_{ij}:

$$N_i(T) = G_i \phi_i + \sum_{j \neq i} G_{ij} f_{(i|ij)} \phi_{ij}$$

Here, the first term corresponds to cells that grow in isolation, modelling the growth in droplets with only one cell-type presents, while the second term models the cell-growth in droplets with a pair of cells from different types. Here, we thus ignore the contribution of 3-cell droplets, because these droplets are rare for the chosen experimental settings. The final cell-type fractions are given by:

$$p_i(T) = \frac{N_i(T)}{\sum_j N_j(T)}$$

Step 4: Determining final cell-type fractions and the likelihood of the final counts

Given these predicted final fractions $\vec{p}(T)$, the probability of the experimentally measured final counts is again multinomially distributed:

$$P(\vec{d}(T), |\vec{p}(T)) = \frac{(\sum_i d_i(T))!}{d_1(T)! d_2(T) \cdots} \prod_i p_i(T)^{d_i(T)},$$

where we should note that the final fractions are directly dependent on the interaction parameters G_{ij} and $f_{(i|ij)}$. To estimate these interaction parameters, we need to obtain a posterior for the parameters by multiplying the probability of the data by prior

distributions $P(G_{ij})$ and $P(f_{(i|ij)})$. Subsequently, we maximise this posterior distribution by jointly optimising all interaction parameters.

The choice of prior distributions

Since G_{ij} is a relative growth factor, we will define its prior in log-space. If we do not have any further prior information on the parameter value, we want to take a uniform prior in log-space, which is the scale prior $P(G_{ij}) \propto 1/G_{ij}$. One can intuitively understand this prior as giving the same weight to the growth factor being close to 0.5 as close to 2. When we do have additional information on the value of G_{ij}, we want to capture this by setting its mean and variance in log-space. The maximum-entropy prior given these constraints is a log-normal distribution:

$$P(G_{ij}) = \frac{1}{G_{ij}\sqrt{2\pi}} exp\left(-\frac{\left(log(G_{ij})-\mu\right)^2}{2\sigma^2}\right).$$

Since the parameters $f_{(i|ij)}$ are fractions, they should only be defined on the unit interval: [0,1]. If we do not have any further prior information, we will thus take the uniform distribution that is 1 over the whole interval. However, if we do have more information, we again want to constrain its mean and variance. In this case, we do this by constraining the mean μ^f and a precisio ν^f. To gain some intuition for the precision, we can also set it by setting the variance σ^2, where $\nu^f = \frac{\mu^f(1-\mu^f)}{\sigma^2} - 1$. The Dirichlet distribution for $f_{(i|ij)}$ is given by:

$$P(f) = \frac{\Gamma(\nu^f)}{\Gamma(\nu^f\mu^f)\Gamma(\nu^f(1-\mu^f))} f^{\nu^f\mu^f-1)}(1-f)^{\nu^f(1-\mu^f)-1}.$$

Determination of the posterior probability interval for the estimated interaction parameters

After computing the maximum posterior value for the parameters, we want to quantify the uncertainty on the estimated parameter values by calculating posterior probability intervals. To determine this interval for a specific parameter, we vary the parameter and re-optimise the other parameters. In particular, let's say we want to determine the uncertainty for the parameter x which has maximum posterior value x^*, and let's denote by $L(x)$ the log-posterior as a function of this parameter, where all other parameters are set to their optimal values. If the posterior were a perfect Gaussian distribution, the standard deviation of the parameter x would coincide with the distance from the optimum where the log-posterior drops by 0.5. Therefore, we approximate the error bar for the parameter x by determining the lower and upper bounds for x at which the log-posterior has dropped by 0.5. Thus, we define the lower bound of the posterior probability interval by demanding:

$$L(x^*) - L(x_{lb}) = 0.5,$$

and similar for the upper bound. If the posterior were Gaussian distributed, this interval would correspond to a 68% probability interval.

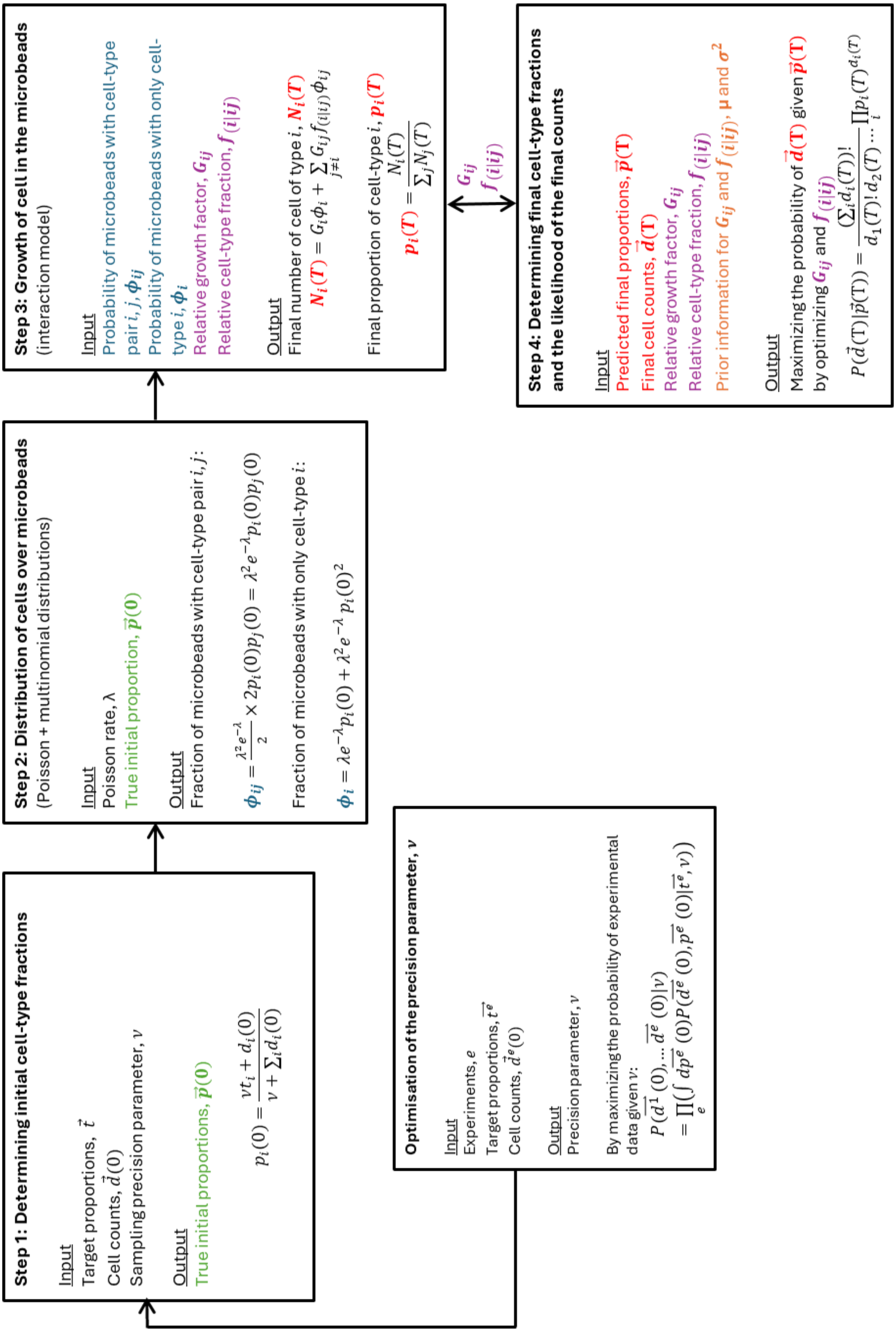

Figure S8: Schematic overview of the model

Prior information for the inference model to predict interactions using experimental data

Table S1: Prior information i.e. means and standard deviations for priors of G_{ij} and $f_{i|ij}$ parameters provided to the inference model to decipher pairwise interactions for the consortium with strains A, B and C. When prior information is available, it is captured in the means and standard deviations of the priors. When no prior information is available, G_{ij} is taken to be uniform in the log-scale and $f_{i|ij}$ to be uniform in the unit interval. This is captured as 'uniform' in the standard deviations for the priors.

**Means for priors of G_{ij} parameters**			
	Strain A	Strain B	Strain C
**Strain A**	0.0001	1	1
**Strain B**	1	0.0001	1
**Strain C**	1	1	0.0001
**Standard deviations for priors of G_{ij} parameters**			
	Strain A	Strain B	Strain C
**Strain A**	0.01	uniform	uniform
**Strain B**	uniform	0.01	uniform
**Strain C**	uniform	uniform	0.01
**Means for priors of $f_{i\|ij}$ parameters**			
	Strain A	Strain B	Strain C
**Strain A**	1	0.5	0.5
**Strain B**	0.5	1	0.5
**Strain C**	0.5	0.5	1
**Standard deviations for priors of $f_{i\|ij}$ parameters**			
	Strain A	Strain B	Strain C
**Strain A**	0.01	uniform	uniform
**Strain B**	uniform	0.01	uniform
**Strain C**	uniform	uniform	0.01

Table S2: Prior information i.e. means and standard deviations for priors of G_{ij} and $f_{i|ij}$ parameters provided to the inference model to decipher pairwise interactions for the

consortium with strains A, B and C. When prior information is available, it is captured in the means and standard deviations of the priors. When no prior information is available, G_{ij} is taken to be uniform in the log-scale and $f_{i|ij}$ to be uniform in the unit interval. This is captured as 'uniform' in the standard deviations for the priors.

**Means for priors of G_{ij} parameters**			
	Strain A	Strain B	Strain C*
**Strain A**	0.0001	1	0.0001
**Strain B**	1	0.0001	1
**Strain C***	0.0001	1	0.0001
**Standard deviations for priors of G_{ij} parameters**			
	Strain A	Strain B	Strain C*
**Strain A**	0.01	uniform	0.01
**Strain B**	uniform	0.01	uniform
**Strain C***	0.01	uniform	0.01
**Means for priors of $f_{i|ij}$ parameters**			
	Strain A	Strain B	Strain C*
**Strain A**	1	0.4	0.5
**Strain B**	0.6	1	0.5
**Strain C***	0.5	0.5	1
**Standard deviations for priors of $f_{i|ij}$ parameters**			
	Strain A	Strain B	Strain C*
**Strain A**	0.01	0.03	uniform
**Strain B**	0.03	0.01	uniform
**Strain C***	uniform	uniform	0.01

SI: 7. Computationally deciphering interactions in larger consortia

Steps for the computational validation:

Step 1: Consortia design

Consortia consisting of four to six members were designed. Two types of consortia were designed: (1) consortia where none of the cell-types could grow independently, i.e., no isolated growth, and (2) consortia where one or two cell types were capable of isolated growth. In scenarios where there is a negative interaction, we assume that both the cell types exhibit isolated growth, and depending on the direction of the interaction, we expect at least one of the cell types to grow.

Based on these consortia design, G_{ij} and $f_{i|ij}$ were defined accordingly. Here, G_{ij} gives the average final number of cells in a microbead that was inoculated with an i, j-pair, and $f_{i|ij}$ denotes the fraction of these cells that is of type i. The eventual number of cells of strain i in this droplet is thus given by $G_{ij}f_{i|ij}$. Examples of these parameter settings are given below.

When a cell-type i exhibited isolated growth, $G_{ij,i=j} = 0.5$ and the corresponding $f_{i|ij,i=j} = 1$. When this cell-type i was co-localised with a cell-type j in the absence of an interaction, the G_{ij}= 0.5, $f_{i|ij} = 1$. In case of positive cross-feeding between cell-types i and j, G_{ij}= 1, $f_{i|ij} = f_{j|ij} = 0.5$.

Step 2: Simulation data

Experimental data was simulated by first generating random target cell-type counts. These cell counts were generated by assuming a total sampled number of cells to be 96 or 1000 cells and by applying random multinomial sampling. This data is collected in count vector $\vec{d}_{sim}(0)$. To account for experimental sampling noise, the initial counts

$\vec{d}_{sim}(0)$ were modelled as a multinomial sample of the strain proportions $\vec{p}(0)$ by using the experiment precision parameter as described in SI:6.

Using the initial strain proportions, the fraction of microbeads with different cell-type compositions (ϕ_{ij}) was modelled as described in SI:6. The number of offspring cells that a cell-combination in a microbead will give rise to is modelled using the parameters G_{ij} and $f_{i|ij}$. Based on the fraction of microbeads with a certain cell-type composition and the model for interactions, the eventual final cell-type frequencies were determined. These frequencies were subsequently converted into final cell-type counts $\vec{d}_{sim}(T)$ by multinomial sampling to account for sampling noise by assuming the total number of sampled cells to be 96 or 1000.

<u>Step 3: Inference model</u>

To estimate these interaction parameters, we need to obtain a posterior for the parameters by multiplying the probability of the simulated data (simulated final cell-type fractions $\vec{p}_{sim}(T)$) by prior distributions Subsequently, we maximise this posterior distribution by jointly optimising all interaction parameters. Mathematical details are described in SI:6.

Note: To account for isolated growth in the inference model, we set the 95% confidence interval for the prior information for the growth terms of the cell types capable of isolated growth between 0.37 and 2.7, because one of the cell types will always grow at least until half the medium depletion. This means that, before incorporating the initial and final count data into the inference model, there is a 95% probability that these predicted growth terms fall within this range.

Step 4: Assessing the prediction

Inference error was computed as the absolute difference between the interaction parameters predicted by the inference model and the corresponding interaction

parameters used in the simulation. The inference error for $f_{i|ij}$ parameters was only computed for all cell-type combinations where the corresponding G_{ij} simulation value was lager than 0.1.

For the data represented in the main text, we set the number of colonies sampled to determine the cell-type fractions to 1000 (Figure 5). To test the effect of the number of colonies to be sampled on inference errors, we reduced this number to 96 (Figure S9). This resulted in overall higher inference errors compared to when the 1000 colonies were sampled.

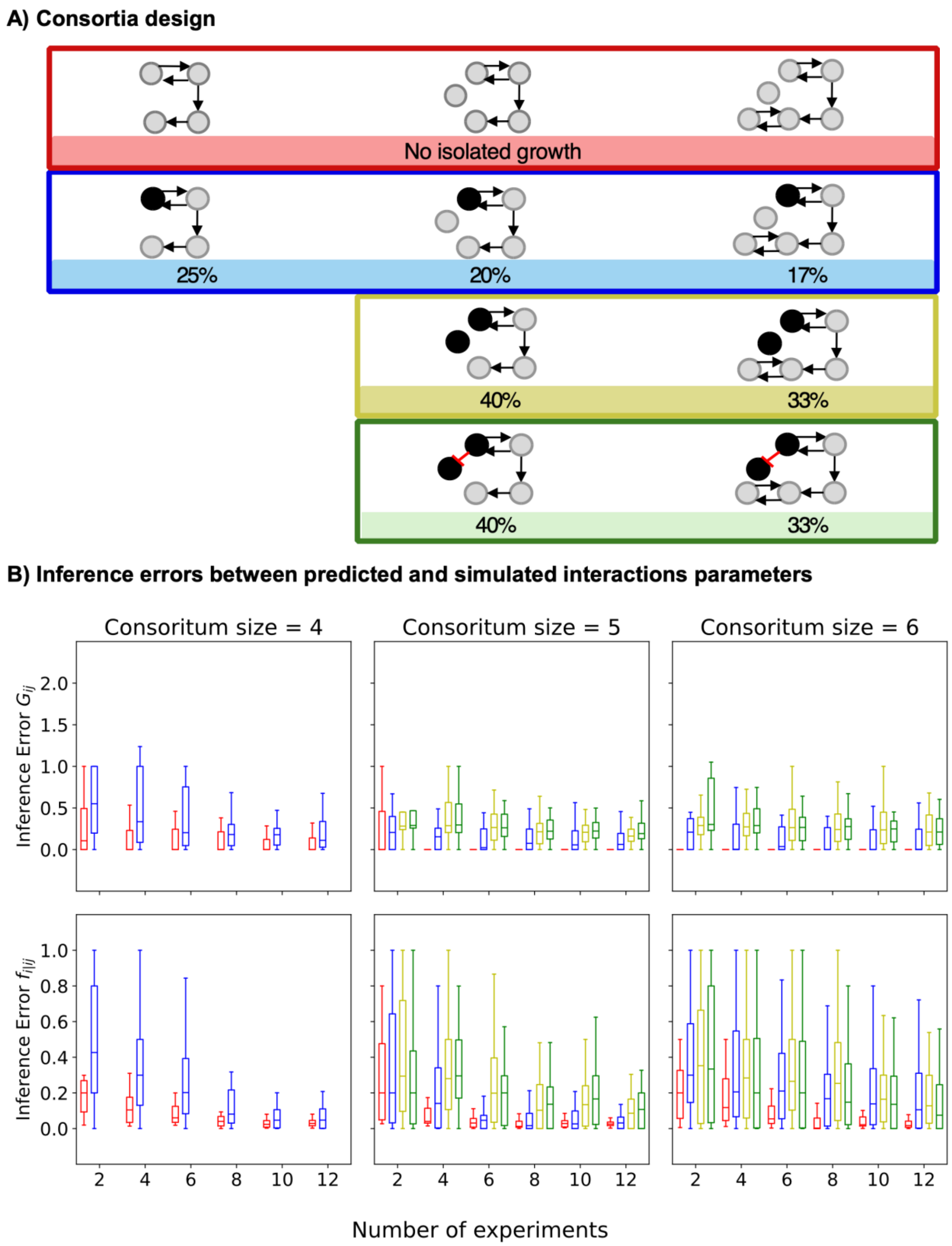

Figure S9: Computational validation to infer interactions in larger consortia, 'high noise' conditions. Inference errors between predicted and simulated interaction parameters. (A) Consortia were designed for simulation; the cell-types that do not grow by themselves, i.e. with no isolated growth, are represented in grey and cell-types that grow by themselves are represented in black. The percentage of consortium members capable of isolated growth is indicated below each consortium. The distribution of inference errors of the growth (G_{ij}) and cell-type fraction terms ($f_{i|ij}$) for different numbers of experiments, where the inference error is the absolute difference between the interaction parameters predicted by the inference model and

the corresponding interaction parameters used in the simulation. For these simulations Poisson rate λ was set to 0.3 and number of colonies to determine cell type abundances was set to 96. The number of experiments corresponds to the number of unique sets of initial cell-type counts. The individual box plots represent the distribution of the inference errors for five random replicates for the same number of experiments. The colours of the box plots correspond to the consortia configurations in panel (A).
